# Spin effects in superfluidity, neutron matter, and neutron stars

**DOI:** 10.1126/sciadv.aef0431

**Published:** 2026-09-16

**Authors:** Armen Sedrakian, Peter B. Rau

**Affiliations:** ^1^ Frankfurt Institute for Advanced Studies, 60438 Frankfurt am Main, Germany.; ^2^ Institute of Theoretical Physics, University of Wrocław, 50-204 Wrocław, Poland.; ^3^ Columbia Astrophysics Laboratory, Columbia University, New York, NY 10027, USA.

## Abstract

We review selected aspects of the interior physics of compact stars, focusing on the microscopic and macroscopic manifestations of spin, magnetic fields, and nucleonic superfluidity and superconductivity. Spin statistics of fermions allow quantum degeneracy pressure to determine the stability and global properties of neutron stars, whose structure depends sensitively on the strong interactions among baryons in dense matter. Using a generic metamodeling framework based on an expansion of the nuclear energy density around the isospin-symmetric and saturation-density limits, we highlight how various lesser-known terms in this expansion affect compact-star observables and review multimessenger constraints on mass, radius, and moment of inertia. The influence of magnetic fields on dense matter is examined, showing that substantial effects in their structure require extremely strong fields, whereas lower fields are sufficient to affect their superfluid phases. At the mesoscopic scale, the coexistence of superfluid and superconducting components features vortex and flux-tube lattices, with pinning and mutual friction processes playing central roles in neutron-star rotational dynamics. We discuss unresolved issues concerning vortex structure, flux-tube configurations, and the origin of pulsar glitches and postglitch relaxation. We also briefly address the possible emergence of deconfined quark phases in compact-star cores, including their color-superconducting properties, as well as the associated vortex structures and magnetic-field responses in such phases.

## INTRODUCTION

The year 2025 marks the centennial of the proposal of electron spin—an intrinsic two-valued form of angular momentum—by Uhlenbeck and Goudsmit (*1*). Their work provided the first clear formulation of spin as an intrinsic property of the electron, building on earlier experimental and theoretical indications, including the anomalous Zeeman effect, the Stern-Gerlach experiment, and early discussions of electron magnetic moments by A. Compton. Originally introduced to explain the puzzling doubling and splitting of atomic spectral lines, the concept of spin soon revolutionized physics across all scales. From atomic structure and chemistry to condensed matter, quantum field theory, and modern quantum technologies, spin has become a cornerstone of our understanding of nature. Although initially met with skepticism, the concept soon found solid theoretical grounding. Pauli formalized its quantum description through his matrices and the exclusion principle (*2*, *3*), while Dirac’s relativistic wave equation (*4*) revealed spin as an inherent and inevitable consequence of relativistic quantum mechanics.

In astrophysics, the impact of spin is especially profound. Unlike ordinary stars, neutron stars are supported not by thermal pressure but by the degeneracy pressure arising from the Pauli exclusion principle and the spin-statistics connection for fermions. In this sense, neutron stars represent nature’s most notable astrophysical manifestation of fermionic spin: city-sized stellar remnants held up against gravity primarily by the quantum property of spin in their constituent neutrons (and, to a lesser degree, other baryons). What began as an ad hoc solution to a spectroscopic puzzle thus became a foundational pillar of modern physics, linking the microscopic structure of elementary particles to the macroscopic stability of stars.

Neutron stars are typically understood as objects composed primarily of nucleonic degrees of freedom, with an admixture of electrons (and muons at sufficiently high densities). They belong to the broader class of compact stars, all of which derive their stability from degeneracy pressure: (i) White dwarfs, characterized by lower average densities and substantially larger radii, are supported by the pressure of a cold, relativistic electron gas; (ii) hybrid and hyperonic stars, which can be regarded as subclasses of neutron stars, may be stabilized (in part) by the degeneracy pressure of cold, dense quark matter or hyperonic components, respectively, located deep within the stellar core and surrounded by an envelope of ordinary nuclear matter; (iii) strange stars are hypothesized to consist of nearly equal numbers of up, down, and strange quarks, with their stability arising from quark degeneracy pressure. These objects remain elusive, with no observational evidence to date supporting their existence. White dwarfs are separated from neutron stars by an instability region in parameter space (e.g., central density versus stellar mass), whereas hybrid stars may or may not form distinct branch(es) relative to neutron (or hyperonic) stars. Strange stars are generally disconnected from other compact-star families.

Thus, the centennial of electron spin is also a moment to reflect on and review its cosmic implications: the stability of compact stars, the fate of massive stellar evolution, and the extraordinary role played by a quantum mechanical principle, first invoked to explain the fine details of atomic spectra, in producing the strongest-gravity, most compact exotic stellar objects in the universe.

This Review provides a concise and up-to-date account of the interior physics of compact stars, with particular emphasis on recent theoretical and observational developments, as well as on the fundamental role of spin in determining their structural and dynamical properties. Neutron-star interiors may contain a significant—and in some cases even dominant—fraction of heavy baryons, such as hyperons and Δ-resonances. These can be viewed as heavier counterparts of nucleons: Hyperons carry strangeness, while Δ-resonances correspond to higher-spin excitations. In what follows, we use the term “neutron stars” to denote objects composed primarily of nucleonic degrees of freedom; however, we comment where appropriate on the modifications due to other degrees of freedom that become relevant.

The “Spin and the EoS of Neutron Stars” section outlines the equation of state (EoS) of neutron-star matter and summarizes current astrophysical constraints on neutron-star mass, radius, and moment of inertia. Several representative numerical examples are presented to illustrate generic trends. The “Spin and magnetic field” section examines the microphysics of strongly magnetized neutron stars, highlighting the influence of spin in such environments, especially the nontrivial consequences of spin degeneracy lifting and Landau quantization. The “Spin and superfluidity in neutron stars” section reviews superfluidity and superconductivity in neutron-star interiors, emphasizing pairing patterns that depend sensitively on the spin configuration of the neutron and proton Cooper pairs in different density regimes. The spin-1 triplet pairing and effects of the magnetic field on the pairing patterns are discussed, together with the emergence of quantum vorticity as a response to rotation and the magnetic field. Last, the “Superfluidity and astrophysics of compact stars” section considers the macroscopic manifestations of superfluidity in neutron stars and their astrophysical implications, focusing on rotational irregularities such as glitches and long-term precession phenomena. This Review concludes with a discussion of pairing, spin, and superfluidity in quark matter in the “Spin, superfluidity, and vorticity in quark matter” section, where we emphasize the analogies with and differences from nucleonic systems rather than presenting a fully independent treatment. Nevertheless, we provide key references to guide the interested reader toward more detailed studies of this topic.

The selection of topics and examples in this review is, naturally, guided by the authors’ own research interests and emphasizes the role of spin, which constitutes the central theme of this work. Furthermore, the bibliography is intended to be illustrative rather than exhaustive, highlighting representative contributions rather than offering a comprehensive survey of the field.

## SPIN AND THE EOS OF NEUTRON STARS

### Spin, quantum statistics, and degeneracy pressure

At the most fundamental level, the spin-statistics connection ensures the existence of compact stars through degeneracy pressure. Neutrons, as spin-1/2 fermions, obey Fermi-Dirac statistics, and their degeneracy pressure counteracts gravitational collapse. Additional baryonic species, such as protons, hyperons, and baryon resonances, also contribute—although typically at a smaller level, depending on the star’s composition. While hyperons, such as nucleons, carry spin 1/2, Δ-resonances have spin 3/2; both are fermions and contribute to the total degeneracy pressure, with their impact entering through different degeneracy factors in the phase-space integrals and from the specifics of their distinct dynamical equations (Dirac equation or Rarita-Schwinger equation) and strong and electroweak interactions with their environment (*5*, *6*).

Although the model of a noninteracting degenerate Fermi gas is unrealistic, it is worthwhile to briefly look at it, first, because it was the first model to describe degenerate compact stars and, second, it exhibits the importance of the spin and Fermi statistics in creating the degeneracy pressure (*7*, *8*). At zero temperature, the picture is such that all the states below the Fermi energy $E_{F,n}$ or the associated momentum $p_{F,n}$ are occupied, whereas all the states above are free. The relativistic spectrum of neutrons $E_{F,n}=\sqrt{m_{n}^{2}c^{4}+p_{F,n}^{2}c^{2}}$, where $m_{n}$ is the (bare or effective) neutron mass and $p_{F,n}$ is the Fermi momentum, is useful to express in terms of the dimensionless relativity parameter for neutrons $x_{n}$ via $E_{F,n}=m_{n}c^{2}\text{cosh}\left(x_{n}/4\right),$ $x_{n}=4{\text{sinh}}^{-1}\left(p_{F,n}/m_{n}c\right).$ Thus, $x_{n}$ encodes the degree of relativism of the neutrons: Small $x_{n}$ corresponds to the nonrelativistic limit, while large $x_{n}$ corresponds to the ultrarelativistic regime. Computing the energy density by integrating the distribution function of neutrons over momentum space up to the Fermi momentum and similarly the pressure using the standard thermodynamics formulas, one finds at zero temperature (*9*)

$$E_{n}=K_{n}\left(\text{sinh}x_{n}-x_{n}\right)$$ (1)

$$P_{n}=K_{n}\left[\frac{1}{3}\text{sinh}x_{n}-\frac{8}{3}\text{sinh}\left(\frac{x_{n}}{2}\right)+x_{n}\right]$$ (2)

where $K_{n}=m_{n}^{4}c^{5}/32\pi^{2}\hbar^{3}$ sets the overall density and pressure scale. It has dimensions of energy density and arises naturally from the phase-space volume of fermions (counting states per unit volume in momentum space with a factor of 2 for spin degeneracy assuming spin-1/2 fermions). Together, Eqs. 1 and 2 describe the EoS of cold, degenerate neutrons. The limiting cases of nonrelativistic $x_{n}\ll 1$ and ultrarelativistic $x_{n}\gg 1$ gas are easily recovered by expanding these equations respectively in $x_{n}$ and $x_{n}^{-1}$, showing the polytropic forms of the EoS in these two limits

$$P_{n}\propto \left\{\begin{array}{ll} E_{n}^{5/3}, & x_{n}\ll 1,\\ E_{n}^{4/3}, & x_{n}\gg 1 \end{array}\right.$$ (3)

Although real neutron-star matter involves strong nuclear interactions, the relativistic Fermi gas model highlights the role of the Pauli principle and spin-statistics in supporting neutron stars against gravity (*8*). Corrections due to interactions are, however, significant. The predictions for the global parameters of the neutron stars by the degenerate ideal gas EoS are unrealistic; in particular, the maximum mass of a sequence of compact stars turns out to be too low and in contradiction to the observations.

### Astrophysical limits

Pulsar timing provides precise neutron-star mass measurements through Keplerian and relativistic parameters of binary systems, including neutron star–neutron star and neutron star–white dwarf pairs. Although neutron star–black hole binaries have been detected in gravitational waves, they have not yet yielded strong constraints on the neutron-star structure. The Shapiro delay method—based on the extra time radio pulses take to traverse the gravitational field of a companion—has been key in measuring the masses of massive pulsars in a binary with a white dwarf (*10*). The first such measurement, for PSR J1614–2230, yielded a mass of $1.908\left(16\right)\;M_{⊙}$ (solar mass) (*11*–*13*). The second, PSR J0348+0432, was found to have $M=2.01\pm 0.04\;M_{⊙}$ using combined timing and optical modeling (*14*). The most massive to date known neutron star, PSR J0740+6620, has a measured mass of $2.08\pm 0.07\;M_{⊙}$ (*15*).

In general relativity, stability requires that stellar mass increase with central density according to the Bardeen-Thorne-Meltzer criterion (*16*), which is equivalent to the requirement that the oscillation modes of the star remain stable on the ascending branch of the mass–central density curve. Measurements of massive pulsars thus firmly establish that the maximum mass of a neutron star should not be lower than the observed one (currently $M≃2\;M_{⊙}$), setting a lower bound on the maximum mass allowed by any viable EoS.

Gravitational-wave observations from binary neutron-star mergers now provide complementary constraints. The LIGO-Virgo detections of GW170817 and GW190425 (*17*, *18*) enabled the measurement of tidal deformability, which relates the induced quadrupole moment $Q_{\mathrm{ij}}$ to the external tidal field $E_{\mathrm{ij}}$ via

$$Q_{\mathrm{ij}}=-\lambda E_{\mathrm{ij}}=-\frac{2}{3}k_{2}R^{5}E_{\mathrm{ij}}$$ (4)

where λ is the tidal deformability parameter, R is the stellar radius, and $k_{2}$ the Love number (*19*, *20*). It is seen that $\lambda \propto R^{5}$, showing its sensitivity to the EoS. Analyses under low-spin priors yielded effective (dimensionless) deformabilities $\tilde{\Lambda }≃300_{-190}^{+500}$ for GW170817 and an upper limit $\tilde{\Lambda }\leq 600$ for GW190425, implying that radii R≃11 to 13.5 km for typical $1.4\;M_{⊙}$ stars (*17*, *18*).

X-ray pulse profile modeling with the orbital NICER instrument has provided independent mass-radius constraints. For PSR J0030+0451, analyses gave M=1.34 to $1.44M_{⊙}$ and R=12.7 to 13.0 km (*21*, *22*), while for PSR J0740+6620, NICER found R=12.4 to 13.7 km (*23*, *24*) and a mass consistent with that obtained from radio-timing observations quoted above. These constraints—such as those inferred from gravitational-wave observations—do not correspond to single data points with 1σ error bars but rather to posterior probability regions in a two-dimensional parameter space (e.g., the mass-radius or tidal deformability planes). Recent reanalyses (combining NICER with XMM-Newton data from 2023 to 2024) suggest radii that are consistent with or slightly smaller than earlier estimates (*25*), with values around R≈12.3±0.6 km, thereby favoring an EoS featuring a relatively steep rise in pressure beyond nuclear density to support massive neutron stars. In summary, combining pulsar timing, gravitational-wave, and NICER measurements jointly constrains the EoS: The maximum mass must exceed $2M_{⊙}$, while the radii of ∼1.4 to $∼2.0M_{⊙}$ stars cluster around ∼12 to 13 km, implying a weak dependence of the radius on the stellar mass.

Binary neutron-star systems containing two pulsars provide an exceptional natural laboratory for testing the predictions of relativistic astrophysics (*26*). These rare systems enable a variety of precision tests of general relativity, including measurements of orbital decay due to gravitational wave emission, Shapiro delay, and relativistic periastron precession. In addition to their role in testing gravity, such systems also offer valuable constraints on the EoS of dense nuclear matter because pulsar timing allows for highly accurate determinations of stellar masses and, potentially, moments of inertia.

A particularly notable example is the double pulsar system PSR J0737–3039, which consists of two active, radio-emitting neutron stars. This system is unique because both stars are detectable as pulsars, allowing an unprecedented level of orbital and timing precision. Over long observational baselines, relativistic effects—especially the periastron advance—can be used to extract the moment of inertia of pulsar A, denoted $I_{A}$, solely from measurable post-Keplerian parameters, without relying on theoretical modeling of the EoS. This possibility was first highlighted by Lattimer and Schutz (*27*), who demonstrated that a measurement of $I_{A}$ to 10% accuracy could directly translate into strong constraints on the neutron-star EoS. Subsequent statistical analyses by Kramer *et al.* (*26*) have derived posterior probability distributions for the component masses and the moment of inertia of pulsar A, yielding the upper bound $I_{A}<3.0\times 10^{45}\;g\;{\text{cm}}^{2}$ at the 90% confidence level. Continued timing observations of PSR J0737–3039 over the coming decade or so are expected to refine the measurement of relativistic parameters to the point where the empirical uncertainty on $I_{A}$ becomes comparable to that obtained from x-ray pulse-profile modeling by the NICER mission. This measurement would represent a major step forward, providing a purely dynamical, model-independent determination of a neutron star’s moment of inertia—and, by extension, a stringent new test of dense-matter physics.

### Equation of state

The role of spin in unpolarized neutron-star matter, which is a good approximation for the magnetic fields typical of ordinary pulsars, *B* ∼ 10^12^ to 10^13^ G, is straightforward: It effectively doubles the phase space available to fermions at a given energy state, corresponding to the spin-up and spindown configurations. From a microscopic perspective, however, the interaction channels described by the partial wave expansion of the nuclear potential obey spin-isospin selection rules, so spin plays a nontrivial role.

In low-density neutron matter, spin-singlet ^1^*S*_0_ interactions between neutrons dominate, as well as similar interactions between protons, which have much lower densities. In contrast, in symmetric nuclear matter, the dominant low-density interaction is the coupled-channel spin-triplet ^3^*S*_1_-^33^*D*_1_ interaction, which is, however, suppressed at the extremely large isospin asymmetries present in neutron stars and enforced by weak interactions, which establish beta-equilibrium. At higher densities, p-wave channels become important; these contain both spin-singlet and spin-triplet contributions, specifically the spin-singlet ^1^*P*_11_ and spin-triplet ^3^*P*_0_, ^3^*P*_1_, and ^3^*P*_2_-^3^*F*_2_ channels, the last of which dominates the pairing interaction between neutrons at densities above the saturation density $\rho_{\text{sat}}$.

To gain a qualitative understanding of these interactions, it is useful to express the energy per baryon of nuclear matter as an expansion in a Taylor series

$$E\left(\chi ,\delta \right)≃E_{\text{sat}}+\frac{1}{2}K_{\text{sat}}\;\chi^{2}+\frac{1}{6}Q_{\text{sat}}\;\chi^{3}+E_{\text{sym}}\left(\chi \right)\delta^{2}+\ldots$$ (5)

with

$$E_{\text{sym}}=J_{\text{sym}}+L_{\text{sym}}\chi +\frac{1}{2}K_{\text{sym}}\chi^{2}+\frac{1}{6}Q_{\text{sym}}\chi^{3}+\cdots$$ (6)

where $\chi =\left(\rho -\rho_{\text{sat}}\right)/3\rho_{\text{sat}}$ and $\delta =\left(\rho_{n}-\rho_{p}\right)/\rho$, with $\rho_{n}$ and $\rho_{p}$ being the neutron and proton densities. We note that Eq. 5, apart from straightforward expansion in density, also corresponds to an expansion in the isospin asymmetry parameter δ, truncated at quadratic order (the parabolic approximation). While this approximation is well justified near symmetric nuclear matter, its validity becomes less controlled for highly neutron-rich matter (δ∼1), where higher-order terms in δ may contribute nonnegligibly. Its use in the neutron-star context is nevertheless supported by empirical evidence from microscopic models, which indicate that the quadratic approximation remains reasonably accurate even at large isospin asymmetry (*28*). The symmetry energy expansion can incorporate hyperons by including the strangeness quantum number as an expansion parameter and redefining the isospin asymmetry parameter (*29*).

The coefficients of the expansion of the symmetric nuclear matter are the binding energy $E_{\text{sat}}$, incompressibility $K_{\text{sat}}$, and the skewness $Q_{\text{sat}}$; the coefficients of the density expansion of $E_{\text{sym}}\left(\chi \right)$ are the symmetry energy $J_{\text{sym}}$, the slope and curvature parameters $L_{\text{sym}}$ and $K_{\text{sym}}$, and the skewness of symmetry energy $Q_{\text{sym}}$. These are defined explicitly as

$$K_{\text{sat}}=9\rho_{\text{sat}}^{2}\frac{d^{2}E\left(\chi \right)}{d\rho^{2}},\;Q_{\text{sat}}=27\rho_{\text{sat}}^{3}\frac{d^{3}E\left(\chi \right)}{d\rho^{3}}$$ (7)

and

$$L_{\text{sym}}=3\rho_{\text{sat}}\frac{{\mathrm{dE}}_{\text{sym}}\left(\chi \right)}{d\rho },\;K_{\text{sym}}=9\rho_{\text{sat}}^{2}\frac{d^{2}E_{\text{sym}}\left(\chi \right)}{d\rho^{2}}$$ (8)

where all derivatives are evaluated at $\rho =\rho_{\text{sat}}$. The expansion (Eq. 5) is rooted in the infinite-matter limit of the Bethe-Weizsäcker nuclear mass formula. Hence, its low-order coefficients are well constrained by experimental data on finite nuclei. In particular, the binding energy per nucleon at saturation, $E_{\text{sat}}≃-16.0\pm 1$ MeV, the nuclear symmetry energy at saturation, $J_{\text{sym}}≃32.0\pm 2$ MeV, and the saturation density itself, $\rho_{\text{sat}}≃0.15\;\text{to}\;0.16\;{\text{fm}}^{-3}$, are among the most reliably determined quantities. Higher-order coefficients in the density expansion, both in the isoscalar (Eq. 7) and isovector (Eq. 8) channels are less constrained by direct experiments. Instead, they have been studied using a combination of statistical Bayesian analyses (*30*–*33*), metamodeling approaches (*34*), and multimessenger observations of compact stars (*35*, *36*). In general, the validity of the Taylor expansion (Eq. 5) is limited to the condition χ<1, which corresponds to densities to a few times $\rho_{\text{sat}}$ depending on the parametrization of density functional for nucleonic models, but this estimate may change if additional degrees of freedom, such as hyperons or Δ-resonances, become relevant or if deconfinement phase transition to quark matter occurs.

Despite considerable progress, $K_{\text{sat}}$ is still estimated within a relatively broad range, $200≲K_{\text{sat}}≲300$ MeV. Heavy-ion collision experiments favor a relatively soft EoS, implying lower $K_{\text{sat}}$, although such conclusions remain model dependent. The skewness parameter $Q_{\text{sat}}$, which characterizes the third derivative of the energy with respect to density, remains largely unconstrained. Estimates in the literature vary widely, typically within $-600≲Q_{\text{sat}}≲1000$ MeV. The above constraints pertain specifically to nucleonic matter. Their applicability to dense matter containing heavier baryons, such as hyperons or Δ-resonances, is uncertain and subject to ongoing investigation (*6*).

### Mass, radius, and moment of inertia

The static properties of compact stars, in the simplest case assuming spherical symmetry (i.e., negligible rotation and weak magnetic fields), are obtained by integrating the Tolman-Oppenheimer-Volkoff equations (*7*, *8*). These equations represent the solution of Einstein’s field equations for a spherically symmetric mass distribution in hydrostatic equilibrium. To facilitate comparison between theoretical predictions and observations, it is often useful to present results in terms of directly observable quantities, such as the stellar mass, radius, moment of inertia, and spin frequency.

**Fig. 1.**
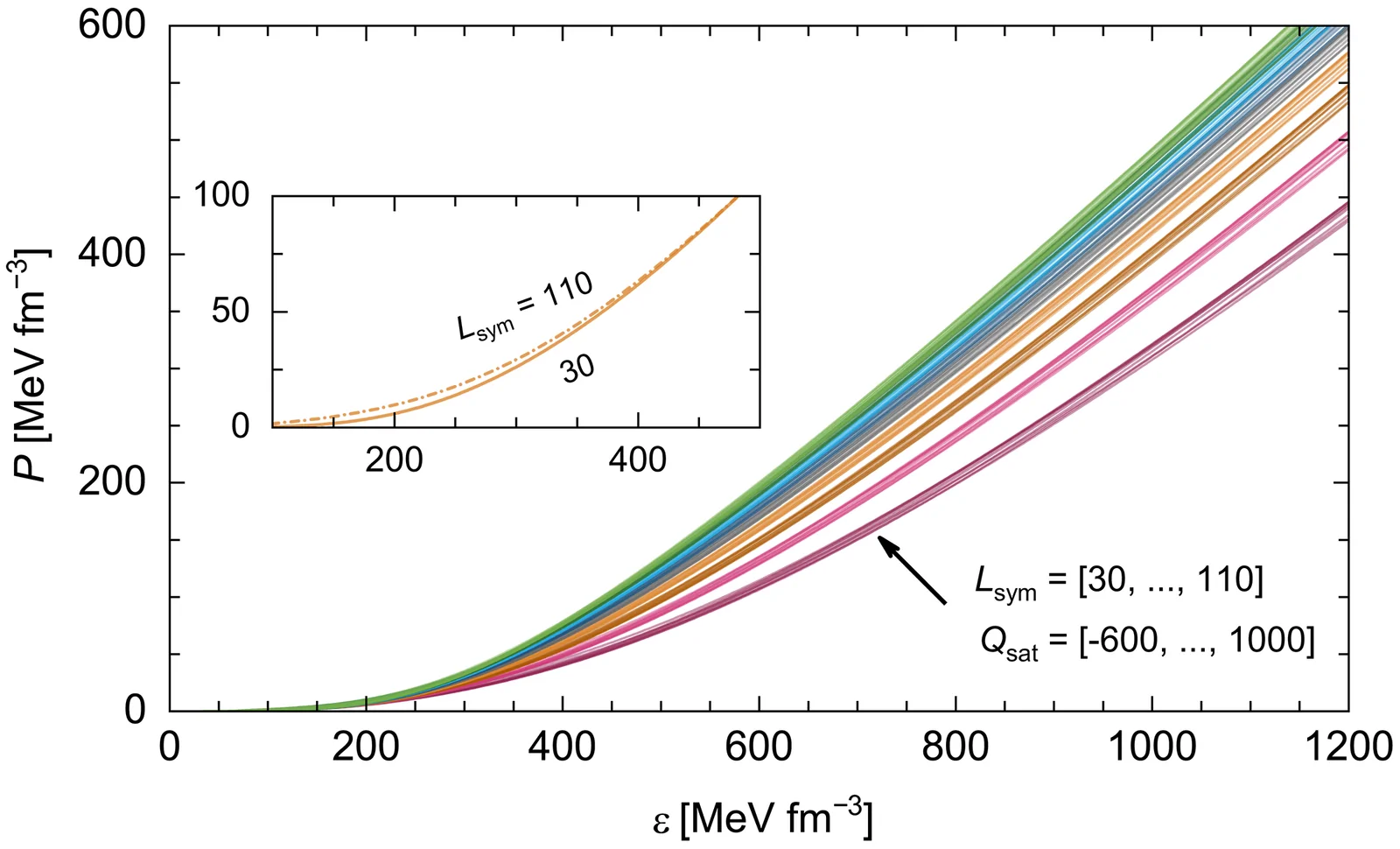
Variation of the nuclear equation of state with $Q_{\text{sat}}$ and $L_{\text{sym}}$ in covariant density functional models. Family of EoS generated using covariant density functional models with the parameters varied over the ranges $Q_{\text{sat}}\in \left[-600,1000\right]$ MeV and $L_{\text{sym}}\in \left[30,110\right]$ MeV (distinguished by color); other parameters of covariant density functional are fixed to that of DDME2 parametrization; for details, see (*37*). The inset highlights the impact of $L_{\text{sym}}$ on the low-density region of the EoS for illustration.

**Fig. 2.**
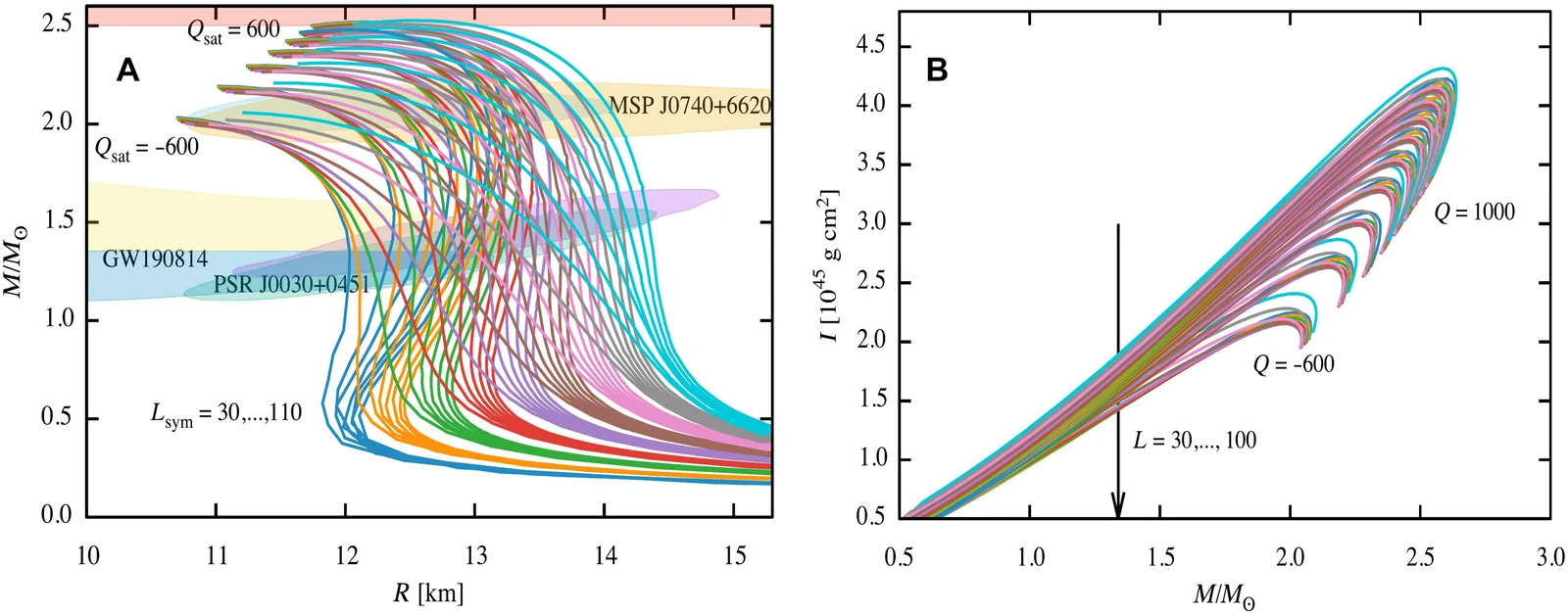
Mass-radius and moment-of-inertia–mass relations for nucleonic equations of state compared with multimessenger astrophysical constraints. (**A**) Mass-radius for nucleonic EoS models corresponding to different $Q_{\text{sat}}$ and $L_{\text{sym}}$ pairs (in mega–electron volts) (*37*). The colored regions indicate the 90% confidence interval (CI) ellipses from the two NICER analyses for PSR J0030+0451 and PSR J0740+6620 (*21*–*24*), the 90% CI regions for the two compact stars involved in the gravitational-wave event GW170817 (*17*), and the 90% CI for the mass of the secondary component in GW190814 (*152*). (**B**) Moment of inertia versus mass for the same EoSs as in (A) for the indicated range of $Q_{\text{sat}}$ and $L_{\text{sym}}$ (in mega–electron volts); the upper limit $I_{A}\leq 3\times 10^{45}$ g cm^2^ at 90% CI on the moment of inertia extracted from PSR J0737–3039 system (shown by vertical arrow) is well satisfied for the collection of EoSs, given that the pulsar mass $m_{A}≃1.34\;M_{⊙}$.

Figure 1 shows representative EoS of neutron-star matter with nucleonic degrees of freedom only constructed from a covariant density functional where the parameters $Q_{\text{sat}}$ and $L_{\text{sym}}$ are varied to illustrate their effect on the pressure of matter (*37*). The corresponding solutions of the Tolman-Oppenheimer-Volkoff equations are shown in Fig. 2A, where the mass-radius relations for neutron-star matter with nucleonic degrees of freedom only are displayed along with the current astrophysical constraints. By systematically varying $Q_{\text{sat}}$ and $L_{\text{sym}}$, one can directly relate microphysical properties of nuclear matter to macroscopic observables, including the stiffness of the EoS, neutron-star radii, tidal deformabilities, and gravitational-wave signatures from binary mergers for a given composition. Of course, this procedure requires additional physical input—namely, assumptions about the composition of the deep interior of the star, whether purely nucleonic, admixed with heavy baryons, or involving phases featuring quark matter.

This approach provides a powerful pathway to constrain nuclear physics models through astrophysical data, establishing a direct connection between dense matter microphysics and observable neutron-star phenomena. For completeness, we show in Fig. 2B the moment of inertia as a function of mass for the same collection of EoS. The upper limit on the moment of inertia of the A pulsar $I_{A}\leq 3\times 10^{45}$ g cm^2^ of PSR J0737–3039 is fully consistent with the entire collection of the EoS, given that its mass was estimated to be $m_{A}≃1.34\;M_{⊙}$.

The possible appearance of heavy baryonic degrees of freedom—such as hyperons and Δ-resonances—in dense matter has important implications for the global properties of neutron stars. Their inclusion is naturally accommodated within covariant density functional approaches, which provide a flexible framework constrained by both laboratory data and astrophysical observations. The onset of hyperons at supranuclear densities generally softens the equation of state, leading to a reduction in the maximum mass of neutron stars—a tension commonly referred to as the hyperon puzzle, given the observation of compact stars with masses above $2\;M_{⊙}$ (*6*, *38*, *39*).

Modern covariant density functional models address this issue by tuning the underlying couplings [e.g., within SU(6) or SU(3) symmetry schemes], thereby enabling sufficiently stiff equations of state that support massive stars while still allowing hyperonic degrees of freedom. In typical scenarios consistent with SU(6) symmetry, hyperons can constitute up to ∼20% of the stellar core composition, with Λ hyperons becoming dominant at high densities. In contrast, models adjusted to reproduce very massive objects tend to suppress the hyperon fraction to only a few percent, effectively yielding nucleonic stars.

Beyond static structure, heavy baryons also influence rotational properties, magnetic configurations, and thermal evolution. In particular, hyperons enable fast cooling via direct Urca processes (*40*), although this can be mitigated by hyperonic pairing, leading to a characteristic mass-dependent cooling behavior (*41*, *42*). Finite-temperature effects, relevant for supernovae and binary neutron-star mergers, further promote the appearance of hyperons at lower densities. Overall, the inclusion of heavy baryons enriches the phenomenology of neutron stars, but their quantitative impact remains sensitive to poorly constrained interactions and many-body effects, making them a key target for future observational and theoretical studies (*43*).

Beyond purely nucleonic and hypernuclear stars, compact stars may also contain deconfined quark matter in their inner cores, leading to so-called hybrid stars. This possibility is particularly interesting because first-order phase transitions in dense matter can leave direct imprints on global stellar properties. If the transition from hadronic to quark matter involves a sufficiently large jump in energy density, the resulting softening and subsequent restiffening of the equation of state can generate new stable branches in the mass-radius diagram. In the simplest case, this gives rise to “twin” configurations, where two stars of the same mass have different radii and internal compositions, one being purely hadronic and the other containing a quark core (*44*–*46*). If the phase structure of dense QCD matter is more intricate, with sequential transitions between distinct quark phases, the mass-radius relation may exhibit an even richer pattern, including additional stable branches and higher-order multiplets (*47*). More generally, even in model-agnostic approaches, the presence of deconfined quark matter can manifest itself through nontrivial features in the EoS, such as a rapid variation of the speed of sound, which are consistent with current astrophysical constraints and can significantly affect masses, radii, and tidal deformabilities (*48*). In this sense, hybrid stars provide a natural extension of the discussion of heavy-baryon degrees of freedom: While hyperons soften the equation of state within the hadronic description, deconfinement introduces qualitatively new phases whose onset and stiffness can reshape the global properties of compact stars in a potentially observable way.

We defer a detailed discussion of pairing, spin, and superfluidity in quark matter to Section 6 after presenting the physics of nucleonic phases. This ordering allows us to use the framework developed for nucleonic superfluids as a basis for comparison, highlighting both the analogies and the key differences that arise in quark matter and providing a coherent entry point to the more specialized literature on this topic.

## SPIN AND MAGNETIC FIELD

For the class of strong–magnetic-field compact stars, known as magnetars, the degeneracy with respect to the spin is lifted by the magnetic-field–spin interaction (*49*, *50*). Magnetars appear as anomalous x-ray pulsars, soft gamma ray repeaters, and as at least some of the fast radio bursts, which can be endowed with exceptionally strong surface magnetic fields, typically in the range of 10^14^ to 10^15^ G. Theoretical models further indicate that the equilibrium configurations of compact stars can support even stronger internal magnetic fields, up to *B* ≲ 10^18^ to 10^19^ G (*51*, *52*). In this range of fields, observable effects on global parameters such as the stellar mass, radius, and moment of inertia occur. This is also a regime, where the star is close to the limit of gravitational stability predicted by virial-theorem arguments and confirmed by general-relativistic stellar models (*51*).

Microscopically, the influence of magnetic fields on the EoS of dense matter becomes significant when the magnetic-field energy associated with charged or magnetized particles approaches the characteristic Fermi energies of the system. Because the Fermi energy increases from the low-density outer layers toward the dense core, stronger magnetic fields are required to significantly modify the EoS at greater depths within the star (*53*–*56*).

In a charge-neutral, beta-equilibrated neutron-star core, the charged constituents—electrons, muons, and protons—are the primary carriers affected by the field. In a magnetic field, the transverse motion of charged particles is quantized into Landau levels (*57*, *58*). For relativistic electrons, assuming a *g*-factor of exactly 2 and a magnetic field in the *z* direction, the energy spectrum takes the form

$$E_{n}\left(p_{z}\right)=\sqrt{p_{z}^{2}c^{2}+m_{e}^{2}c^{4}\left(1+2n\frac{B}{B_{c,e}}\right)}$$ (9)

where n=0,1,2,… labels the Landau level, $p_{z}$ is the momentum along the magnetic field, and $B_{c,e}$ is a critical field defined below. Correspondingly, at zero temperature, the electron number density becomes

$$n_{e}=\frac{\mathrm{eB}}{2\pi^{2}\hbar c}\sum_{n=0}^{n_{\text{max}}}g_{n}p_{F,n}$$ (10)

where $g_{n}=2-\delta_{n0}$ is the spin degeneracy factor, $p_{F,n}$ denotes the Fermi momentum along the magnetic-field direction for the nth Landau level, and $n_{\text{max}}=\left\lfloor p_{F,0}^{2}B_{\text{ce}}/\left(2m_{e}^{2}c^{2}B\right)\right\rfloor$ is the highest occupied Landau level.

Relativistic effects associated with Landau quantization become important once the cyclotron energy, eℏB/(mc), becomes comparable to the particle’s rest energy ${\mathrm{mc}}^{2}$. This defines a critical field strength for each particle species. For electrons, the critical field is

$$B_{c,e}=\frac{\hbar }{e\lambda_{e}^{2}}=4.414\times 10^{13}\;G$$ (11)

where $\lambda_{e}=\hbar /\left(m_{e}c\right)≃400\;\text{fm}$ is the Compton wavelength of the electron. For protons, the critical field is much higher, $B_{c,p}∼10^{20}\;G$, and likely exceeds the hydrostatic stability limit.

Quite generally, Landau quantization reduces the phase space available for charged particles by confining them to a limited number of occupied Landau levels, especially when $B≳B_{c,e}$ and only a few levels are populated. This modification of the density of states alters the pressure and energy density, typically leading to a softening of the EoS. In addition, it affects the composition of matter through changes in the charge fraction $Y_{Q}\left(n_{B}\right)=n_{Q}/n_{B}$, where $n_{B}$ is the baryon density and $n_{Q}$ is the net charge density carried by all charged constituents, such as protons, electrons, muons, or, in the case of deconfined matter, quarks. This quantity characterizes how the electric charge content evolves with density under the constraints of charge neutrality and beta equilibrium.

The underlying reason is that the modified density of states for electrons (and, at sufficiently high fields, other charged species) alters the conditions of charge neutrality and beta equilibrium. As a result, the equilibrium proton and lepton fractions can deviate from their field-free values, particularly at low densities where Landau level quantization is most pronounced. These changes can, in turn, influence macroscopic stellar properties, including the maximum mass and radius, and are therefore particularly relevant for modeling strongly magnetized neutron stars (magnetars); for a broad review of these aspects, see (*50*).

In the same field-strength range, additional effects arise from the anomalous magnetic moments of nucleons. Although neutrons are electrically neutral, their intrinsic magnetic dipole moment couples directly to the magnetic field and contributes to both the energy density and the composition of matter. The corresponding energy shift associated with this coupling is of order $|\kappa_{p}+\kappa_{n}|B$, which modifies the baryon single-particle energies and, consequently, the conditions for beta equilibrium in sufficiently strong magnetic fields. The experimentally determined anomalous magnetic moments of protons and neutrons are

$$\kappa_{p}=\mu_{N}\left(\frac{g_{p}}{2}-1\right),\;\kappa_{n}=\mu_{N}\frac{g_{n}}{2}$$ (12)

where $\mu_{N}=e\hbar /\left(2m_{p}\right)$ is the nuclear magneton and $g_{p}=5.58$ and $g_{n}=-3.82$ are the Landé *g*-factors for the proton and neutron, respectively.

More explicitly, the single-particle spectrum for the baryons acquires a spin-dependent splitting in the presence of a magnetic field. Because the protons are Landau quantized similarly to the electrons, their spectrum differs from that of the neutrons in this way in addition to $\kappa_{p}\neq \kappa_{n}$: These spectra are, ignoring nuclear interactions (*52*)

$$E_{p,s}\left(p_{z}\right)=\sqrt{p_{z}^{2}c^{2}+{\left[M_{p,n,s}\left(B\right)c^{2}+s\kappa_{p}B\right]}^{2}}$$ (13)

$$M_{p,n,s}\left(B\right)=m_{p}\sqrt{1+2\left(n+\frac{1}{2}+\frac{s}{2}\right)\frac{B}{B_{c,p}}}$$ (14)

$$E_{n,s}\left(p_{z},p_{⊥}\right)=\sqrt{p_{z}^{2}c^{2}+{\left[\sqrt{m_{n}^{2}c^{4}+p_{⊥}^{2}c^{2}}+s\kappa_{n}B\right]}^{2}}$$ (15)

where for s=±1, n=0,1,2,… in the definition of $M_{p,n,s}\left(B\right)$ and $p_{⊥}$ is the momentum in the plane perpendicular to the magnetic field. The spin-dependent term lifts the degeneracy between spin-up and spindown states and leads to a partial spin polarization of the baryonic component. This effect becomes appreciable when the magnetic energy scale $\kappa_{b}B$, b=p,n, is comparable to the Fermi energy, which typically requires fields $B≳10^{17}\;\text{to}\;10^{18}\;G$. As a result, both the neutron and proton populations can become spin polarized, altering the energy density and pressure, as well as shifting the beta-equilibrium condition through modified chemical potentials.

For comparison, the effect of the magnetic field on electrons is qualitatively different and typically much stronger at lower field strengths due to Landau quantization. As discussed above, the electron single-particle spectrum becomes discretized in the plane perpendicular to the field, see Eq. 9, with a corresponding reduction of the available phase space when only a few Landau levels are occupied. This leads to oscillatory behavior in thermodynamic quantities as a function of density or magnetic-field strength, known as de Haas–van Alphen oscillations, and significantly modifies the electron chemical potential.

Together, these effects—Landau quantization for charged particles and spin polarization from anomalous magnetic moments for baryons—alter the composition of matter, including the charge fraction $Y_{Q}\left(n_{B}\right)$, by modifying the beta-equilibrium condition and the relative populations of particle species. While Landau quantization dominates at relatively lower field strengths $B≳10^{13}\;\text{to}\;10^{14}\;G$, the anomalous magnetic moment effects become important only at much higher fields, characteristic of the most strongly magnetized neutron stars.

For typical neutron-star core densities, nucleon Fermi energies lie in the range of a few to several tens of mega–electron volts above the rest mass energy. Therefore, once $B/B_{c,e}≳10^{5}$, the anomalous magnetic moment contribution becomes comparable to these energies, and their impact on matter composition and pressure cannot be neglected. In this extreme regime, the magnetic coupling of nucleon spins leads to complete spin polarization of neutrons. The alignment of neutron spins enhances the pressure contribution from the neutron sector, producing an overall stiffening of the EoS that counteracts and eventually dominates the softening induced by Landau quantization.

Thus, at the highest magnetic-field strengths relevant for compact stars, the net effect on the EoS results from a competition between two mechanisms: (i) Landau quantization, which reduces the pressure by restricting charged particles to lower Landau levels, and (ii) spin polarization via anomalous magnetic moments, which increases the pressure through magnetization of the neutron component. The balance between these two effects determines whether the EoS becomes softer or stiffer in the presence of extreme magnetic fields.

However, because the magnetic field provides additional support against gravity through both the Lorentz force and the electromagnetic stress energy, self-consistent solutions of Einstein-Maxwell theory show that a star with the same baryon number can sustain a larger gravitational mass and radius than its unmagnetized counterpart (*54*, *55*, *59*). The magnitude of this effect depends sensitively on the magnetic-field geometry and the underlying EoS: It is most pronounced for globally ordered (with significant poloidal) field configurations.

The current understanding of the stability and internal structure of magnetars suggests that mixed magnetic-field configurations, often referred to as twisted-torus geometries, can provide long-term stability over secular timescales of millions of years, comparable to the active lifetimes over which neutron stars sustain their strong magnetic fields.

**Fig. 3.**
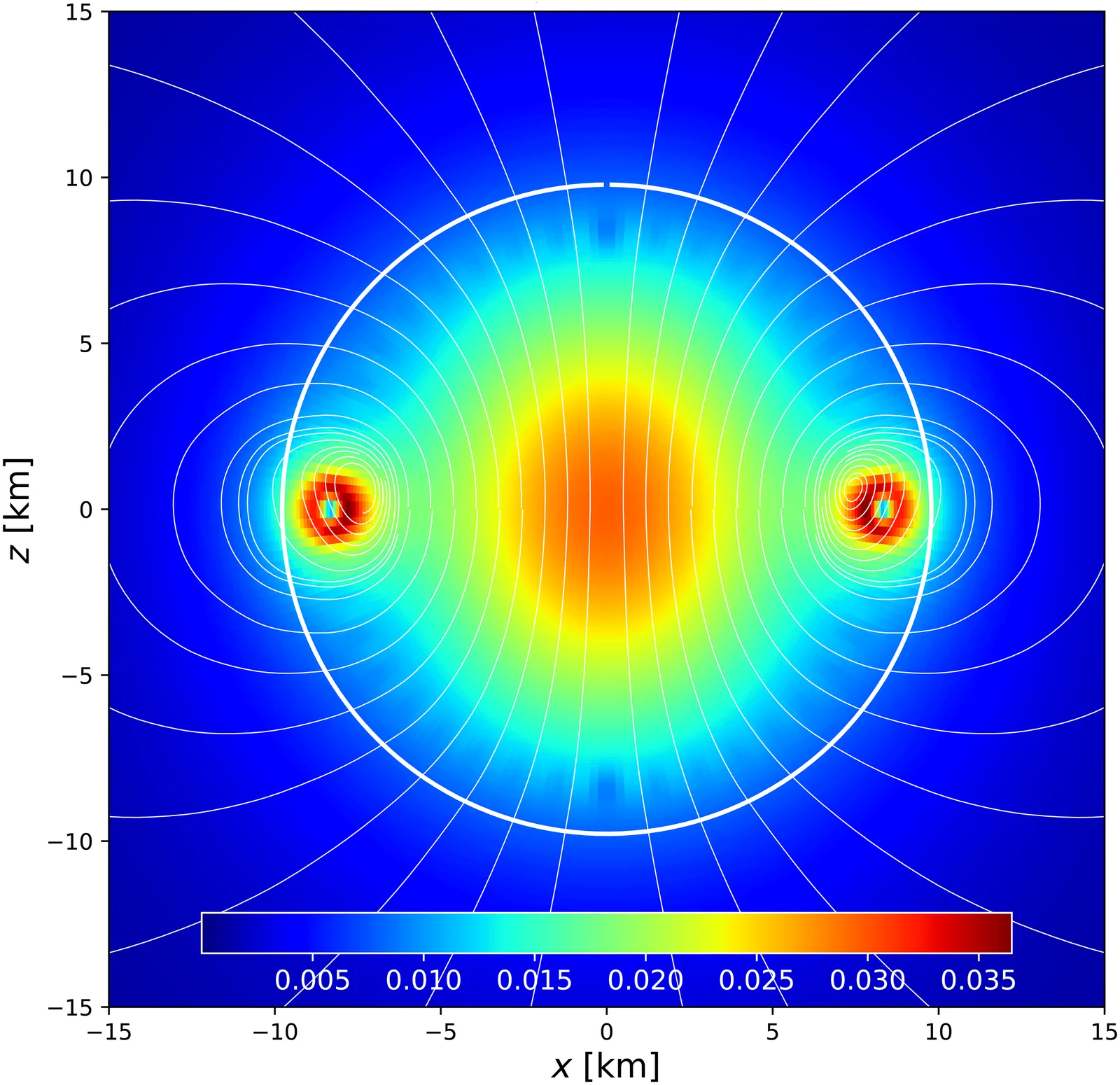
Twisted-torus magnetic-field structure in a magnetar showing coexisting poloidal and toroidal components. Spatial structure of a twisted-torus magnetic field, illustrating the coexistence of poloidal and toroidal components in a magnetar obtained with the XNS solver (*153*). The color bar shows the magnetic-field strength in units of 10^17^ gauss. The white lines trace the dipolar (poloidal) field component, while the toroidal component is confined to the darker equatorial regions.

Purely poloidal or purely toroidal field configurations are known to be unstable to various magnetohydrodynamic instabilities (e.g., kink or Tayler instabilities), whereas a combined configuration can achieve a stable equilibrium by mutually stabilizing these components. In a twisted-torus configuration, the poloidal field threads the star and extends smoothly into the exterior, determining the large-scale dipolar structure observed electromagnetically, while the toroidal component is predominantly confined to closed–field-line regions in the stellar interior, where it can reach strengths exceeding that of the poloidal field; see Fig. 3. These configurations are supported by numerical solutions of the coupled Einstein-Maxwell equations for magnetized, self-gravitating fluids and represent equilibria consistent with both relativistic gravity and realistic equations of state (*59*, *60*). The presence of a substantial internal toroidal field also has important implications for the star’s deformation, stability, and potential gravitational-wave emission.

More generally, the internal magnetic-field configuration of neutron stars is still not well constrained. While global models (e.g., mixed poloidal-toroidal field configurations) exist, they are subject to uncertainties related to stability, composition, superconductivity, and coupling between different components. Direct observational constraints probe the external dipolar field primarily, whereas the internal field strength and geometry—particularly in the core—remain largely uncertain. Consequently, magnetic-field effects can be incorporated at a qualitative or phenomenological level, but a fully self-consistent microphysical and macroscopic description is still an open problem.

At high densities, spin polarization effects can affect the phase structure of the baryonic matter. An extreme but uncertain case is the ferromagnetic phase transition, where the interaction causes a spontaneous transition to a polarized state even in the absence of the magnetic field. Both qualitative and quantitative evidence show that the threshold densities for ferromagnetic or highly spin-polarized phases lie generally at several times nuclear saturation density. The precise threshold depends on the nuclear interaction model, external field, and temperature, with some models showing no evidence for such transition (*61*, *62*). Furthermore, hyperons and quark matter appear at high densities and could preempt ferromagnetic phases. In addition, while pure neutron matter may or may not show ferromagnetic behavior, stellar matter must be subject to electric charge neutrality, β-equilibrium (*63*), and one needs to take into account magnetic screening or shielding by superconductivity (*64*), all of which may alter viability of the ferromagnetic phase.

Landau quantization of charged particles in a strong magnetic field affects several key microphysical processes in neutron stars. In the core, it modifies neutrino emissivity and shifts the threshold density for the direct Urca reaction (*65*, *66*) and introduces Shubnikov–de Haas oscillations in transport coefficients (*67*–*69*). While in the core of a neutron star, where multiple fermion species are Landau quantized, the EoS and other observables are not strongly altered for physically relevant field strengths $B\leq 10^{18}$ G (*52*), in the less-dense crust, these quantization effects are significant. This is especially true within the outer envelope with densities $\rho ≲10^{10}$ g/cm^3^, where quantization effects enhance the thermal conductivity parallel to the magnetic field. This generally results in a higher effective surface temperature for a given envelope-crust boundary temperature for typical mixed dipole-toroidal field configurations (*68*, *70*).

Landau quantization can alter the equilibrium nuclear composition and shift the neutron drip line to higher or lower densities, depending on the field strength (*71*). Furthermore, the quantization of electrons and other charged fermions gives rise to de Haas–van Alphen oscillations in the differential magnetic susceptibility of matter. These oscillations can, in turn, induce the formation of diamagnetic domains, introducing additional anisotropies in the crustal structure (*72*–*74*). They can also enhance the dissipation of the magnetic field through the generation of small-scale field features of size

$$\ell ∼\mathrm{eB}\hbar c\;\mu_{e}\left(\frac{\mathrm{dz}}{d\mu_{e}}\right)∼\frac{230}{g_{14}\mu_{e10}}\left(\frac{B}{10^{14}G}\right)\;\text{cm}$$ (16)

for electron chemical potential $\mu_{e10}$ in units of 10 MeV and gravitational acceleration $g_{14}$ in units of 10^14^ cm s^−2^ (*75*). This is generally much smaller than the large-scale field with length scale of the order of the stellar radius *R* ∼ 10 to 13 km or crust thickness ∼0.1*R*.

## SPIN AND SUPERFLUIDITY IN NEUTRON STARS

### Pairing channels

**Fig. 4.**
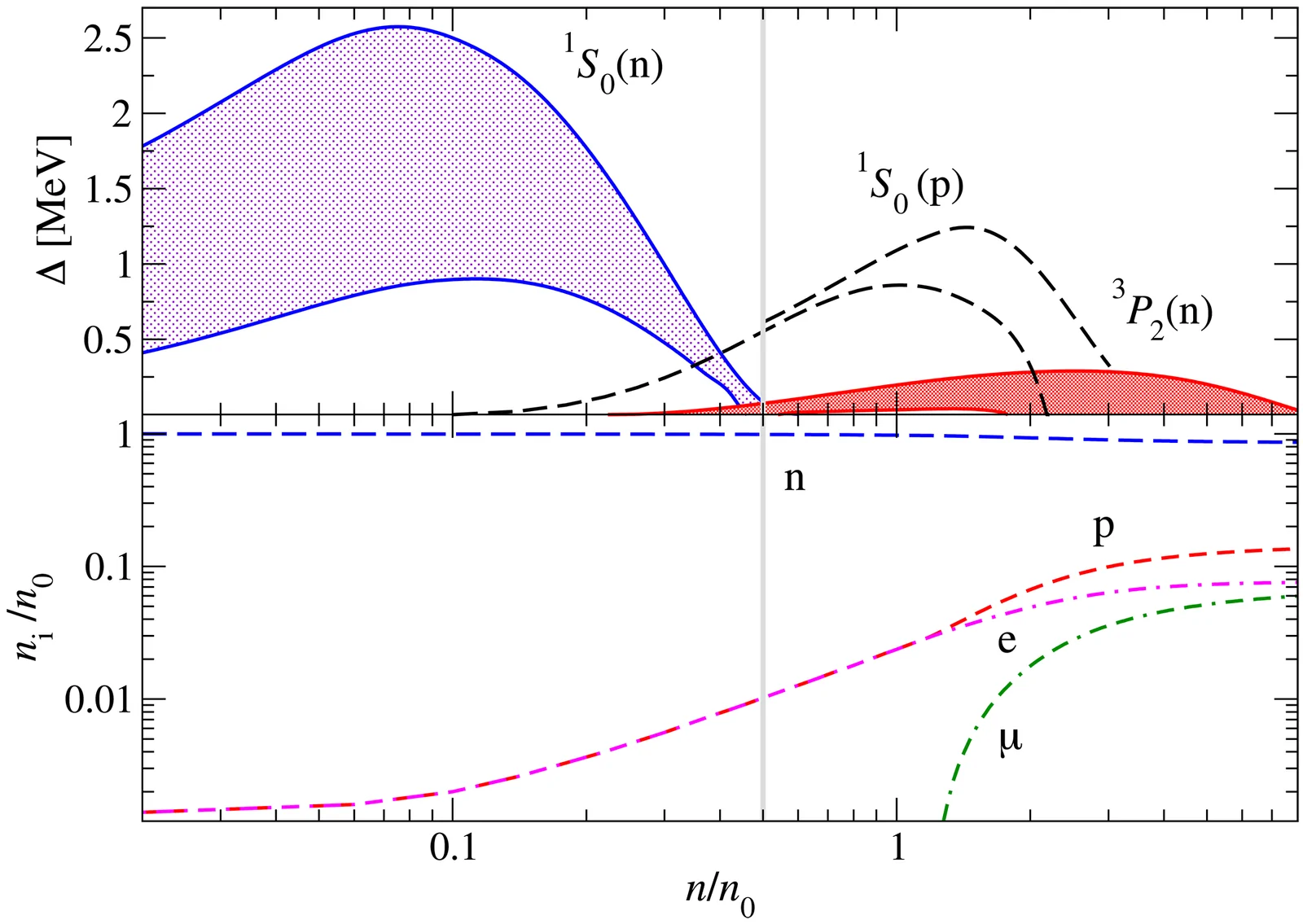
Density dependence of neutron and proton pairing gaps and corresponding particle composition in the neutron-star core. Top: Pairing gaps as functions of baryon density (in units of nuclear saturation density *n*_0_) for neutrons in the ^1^*S*_0_ (solid lines) and ^3^*P*_2_-^3^*F*_2_ (dash-dotted lines) channels and for protons in the ^1^*S*_0_ channel (dashed lines). The shaded areas show the upper and lower bounds on neutron gaps; the upper and lower dashed lines show the same for proton s-wave gap; see (*76*) and references therein. Bottom: Particle composition of the neutron-star core at *T* = 0, corresponding to the conditions used in computing the pairing gaps. The vertical line marks the crust-core interface at *n*/*n*_0_ = 0.5.

Microscopically, spin determines the pairing patterns of fermions, leading to superfluidity and superconductivity within the stellar interior [see (*76*) and references therein]. Figure 4 shows the typical arrangement of superfluid and superconducting phases in the interior of a neutron star for a given composition. Neutrons in the crust and outer core are expected to pair predominantly in the spin-singlet ^1^*S*_0_ channel, while in the higher-density inner core they form pairs in the spin-triplet ^3^*P*_2_ channel. Protons, which are present at the few-percent level, pair mainly in the ^1^*S*_0_ state and form type-II superconducting condensates at low densities and type-I superconducting condensates at high densities. The interplay of these spin-dependent condensates governs glitch phenomena, vortex–flux-tube interactions, neutrino emissivities, and the overall thermal and rotational evolution of neutron stars. Moreover, strong magnetic field restructures the phase diagram of superfluids and superconductors: Proton superconductivity may be suppressed at fields above the critical value, while the neutron ^3^*P*_2_-^3^*F*_2_ superfluid phases can acquire spin-aligned order parameters. These effects propagate into transport processes, including thermal and electrical conductivities, viscosities, and neutrino emission rates, all of which are sensitive to spin alignment and field strength. The spin-magnetic interaction thus emerges as a critical ingredient in the microphysics of magnetars and in determining their observational signatures (*50*).

The role of spin in pairing in nuclear and neutron-star matter can be understood from the partial-wave analysis of nucleon-nucleon (*NN*) scattering. Two-nucleon states are labeled using standard spectroscopic notation ${}^{2S+1}L_{J}$, where L=0,1,2,… corresponds to S,P,D,… waves, S=0,1 indicates singlet or triplet spin states, and J is the total angular momentum. The Pauli principle requires that the total wave function, including spin and isospin, be antisymmetric, which imposes the condition L+S+T odd.

At low energies, *L* = 0 states dominate, giving rise to the ^1^*S*_0_ and ^3^*S*_1_-^3^*D*_1_ coupled channels. In neutron-rich matter, where neutrons dominate, only T=1 pairing is allowed, so the attractive ^3^*S*_1_-^3^*D*_1_ channel is forbidden. In contrast, symmetric nuclear matter can support T=0 pairing in this channel. At asymptotically low densities, this may lead to a transition to a Bose-Einstein condensate of deuterons; however, higher-order clustering effects are also expected.

Theoretical calculations of pairing are largely based on phase-shift–equivalent nucleon–nucleon potentials, such that the dominance of a given channel is constrained by experimental scattering phase shifts and, in the case of the ^3^*S*_1_-^3^*D*_1_ channel, by the existence of the deuteron bound state. However, the relative strength of different pairing channels can be significantly modified by many-body effects, which may either enhance or reduce the effective attraction and thus alter the resulting pairing gap. In particular, medium polarization effects are known to play an important role, yet their quantitative impact remains uncertain, and no clear consensus has been reached regarding their magnitude (*76*).

In neutron-star matter, with neutron fractions around 95% at saturation and gradually decreasing at higher densities, T=1 pairing dominates. Neutron pairing occurs in the ^1^*S*_0_ channel up to slightly above the crust-core interface, while proton pairing in the same channel persists to higher densities due to their lower Fermi energies. At higher energies, the ^3^*P*_2_-^3^*F*_2_ coupled partial wave becomes the dominant T=1 channel, while at lower-energy (and, thus, densities) ^3^*P*_0_ and ^3^*P*_1_ channels are subdominant or repulsive.

### Spin-1 pairing

The ^3^*P*_2_-^3^*F*_2_ pairing in neutron matter is of major phenomenological importance because the neutron fluid fills most of the stellar core. This form of triplet, odd-parity pairing introduces several new features compared with ^1^*S*_0_ and ^3^*S*_1_-^3^*D*_1_ pairing (*77*). Different magnetic substates can compete, while strong spin-orbit and tensor couplings mix the ^3^*P*_2_ and ^3^*F*_2_ waves.

The microscopic treatment begins with a partial-wave expansion of the pairing interaction and a decomposition of the gap into angular-momentum components. Because the gap equation is nonlinear, these components are coupled. A common simplification is to average over angles, reducing the problem to a one-dimensional integral equation. In most cases, a single channel dominates, but tensor forces couple the p- and f-waves, leading to a system of coupled equations similar to that found in the ^3^*S*_1_-^3^*D*_1_ channel at low densities and near isospin-symmetric nuclear matter. Quantitative predictions for the ^3^*P*_2_-^3^*F*_2_ pairing gap depend sensitively on three-nucleon (3N) forces, which become increasingly important as the density increases. Calculations using the Argonne *V*_18_ potential with the Urbana UIX 3N interaction (*78*) or the Bonn-B interaction, including 3N forces (*79*), typically yield a maximum gap of about 0.5 MeV; see Fig. 4. However, beyond mean-field Bardeen-Cooper-Schrieffer (BCS) theory, effects such as wave-function renormalization can reduce this value by an order of magnitude; see (*80*). The gap is also strongly sensitive to the choice of 3N interaction and emphasizes the need for consistency between 2N and 3N forces (*81*). Neutron ^3^*P*_2_-^3^*F*_2_ superfluidity is of broad and interdisciplinary relevance, as it provides a concrete link between the microphysics of dense nuclear matter in neutron stars and a wider class of systems exhibiting topological quantum order. In particular, its anisotropic, spin-triplet pairing and associated symmetry-breaking patterns closely parallel those realized in superfluid ^3^He, unconventional (e.g., heavy-fermion) superconductors, and engineered ultracold atomic gases, making it a valuable platform for exploring topological phases, collective excitations, and quantum vortices in different areas of physics.

At even higher densities, isospin-symmetric nuclear matter favors ^3^*D*_2_ pairing, but increasing neutron-proton asymmetry drives a transition to ^3^*P*_2_-^3^*F*_2_ pairing. Because even a small isospin imbalance destroys d-wave pairing (*82*), this state can exist only if the deep cores of neutron stars contain nearly symmetric matter, for example, when $K^{-}$ condensation sets in.

### Suppression of pairing by magnetic field

Physical phenomena in dense, strongly interacting matter that depend on the quasiparticle spectrum near the Fermi surface are influenced by magnetic fields of order *B* ∼ 10^16^ to 10^17^ G, which are several orders smaller than those required to affect the EoS, as argued in Section 3. This is particularly relevant for nucleonic pairing and for neutrino radiation processes, which dominate the cooling of compact stars. The field interacts with neutron or proton spins, creating an imbalance between spin-up and spindown particles, which suppresses Cooper pairing because not all spin-up particles have available spindown partners (*83*). This Pauli paramagnetic suppression affects both neutron and proton condensates but is the dominant mechanism for neutrons. Proton pairing, in contrast, is more sensitive to weaker fields through a different mechanism: the Larmor motion of charged protons in the magnetic field, arising from their charge-field interaction (*84*, *85*).

BCS superconductors are characterized by three length scales: (i) the London penetration depth δ, (ii) the coherence length ξ, and (iii) the interparticle distance d. In neutron stars, the condensates satisfy the condition d≪δ,ξ, corresponding to the weak-coupling BCS regime. The ratio of the first two scales defines the Ginzburg-Landau parameter $\kappa_{\text{GL}}=\delta /\xi$ (*86*), which determines the type of superconductivity: $\kappa_{\text{GL}}<1/\sqrt{2}$ corresponds to type I, while $\kappa_{\text{GL}}>1/\sqrt{2}$ indicates type II.

In type-II superconductors, magnetic flux penetrates via quantized vortices carrying flux $\phi_{0}=\text{πℏ}c/e$, whereas in type-I superconductors, the field is either expelled or the matter is split into macroscopic domains of normal and superconducting domains, if the expulsion is ineffective. Combining δ and ξ with $\phi_{0}$ defines three characteristic magnetic fields

$$H_{c1}≃\frac{\phi_{0}}{\delta^{2}},\;H_{\text{cm}}≃\frac{\phi_{0}}{\text{ξδ}},\;H_{c2}≃\frac{\phi_{0}}{\xi^{2}}$$

For type-II superconductors ($\kappa_{\text{GL}}\geq 1/\sqrt{2}$), fluxtubes exist in the field regime $H_{c1}\leq H_{\text{cm}}\leq H_{c2}$: Specifically, above $H_{c1}$, the formation of a single flux tube (Abrikosov vortex) becomes energetically favorable; at $H_{\text{cm}}$—the thermodynamic critical field—the energy densities of the superconducting and normal states are equal; last, $H_{c2}$ corresponds to the field at which superconductivity vanishes because of the overlap of vortex cores; thus, for $H>H_{c2}$, superconductivity of protons is destroyed.

The suppression of pairing in the ^1^*S*_0_-wave neutron condensate differs from that in the proton condensate because charge-neutral neutrons interact with the *B*-field only via their spin magnetic moment (Pauli paramagnetism). The corresponding critical field for destruction of pairing by spin polarization is by an order of magnitude larger (*83*). In the neutron star’s core, neutron pairing shifts to the ^3^*P*_2_-^3^*F*_2_ channel, which involves total spin-1 pairs; in this case, the magnetic field does not destructively affect the internal spin structure (*87*–*89*). As density increases and the proton gap is diminished, their coherence length increases, and eventually the condition for proton type-I superconductivity $\kappa_{\text{GL}}\leq 1/\sqrt{2}$ is satisfied. For fields $H>H_{\text{cm}}$, the matter is in the normal state; otherwise, a (local) Meissner expulsion of the field and breakup into normal-superconducting domains occurs.

Quite generally, the absence or reduction of neutron superfluidity affects many crustal microphysical properties, including neutrino emissivity, transport, thermal relaxation, and dynamical coupling times, which, in turn, influence the damping of oscillations and the interpretation of glitches and antiglitches. While Pauli paramagnetism also applies to s-wave proton pairs, proton pairing is suppressed by the diamagnetic effect associated with the curvature of the proton trajectories in a magnetic field at lower fields, as discussed above; see also (*50*).

### Quantum vorticity and mutual friction

The study of vorticity in nuclear systems is motivated by the rotation of neutron stars, where neutrons form a neutral superfluid that rotates via an array of quantized vortices (*90*). While vortex states in finite nuclei have been conjectured, the condensate coherence length is comparable to or larger than nuclear radii, and one cannot speak of vortices in the usual sense. In contrast, vorticity in neutron stars shares many features with laboratory superfluids and superconductors, including Bose-condensed ^4^He, fermionic ^3^He, and ultracold atomic gases (*91*).

In neutron stars, rotation at angular velocity Ω generates neutron vortices with density

$$n_{n}^{\left(V\right)}=\frac{2\Omega }{\kappa },\;\kappa =\frac{\text{πℏ}}{m_{n}^{*}}$$ (17)

where $m_{n}^{*}$ is the neutron effective mass while type-II proton superconductivity produces electromagnetic vortices with density

$$n_{p}^{\left(V\right)}=\frac{B}{\phi_{0}}$$ (18)

**Fig. 5.**
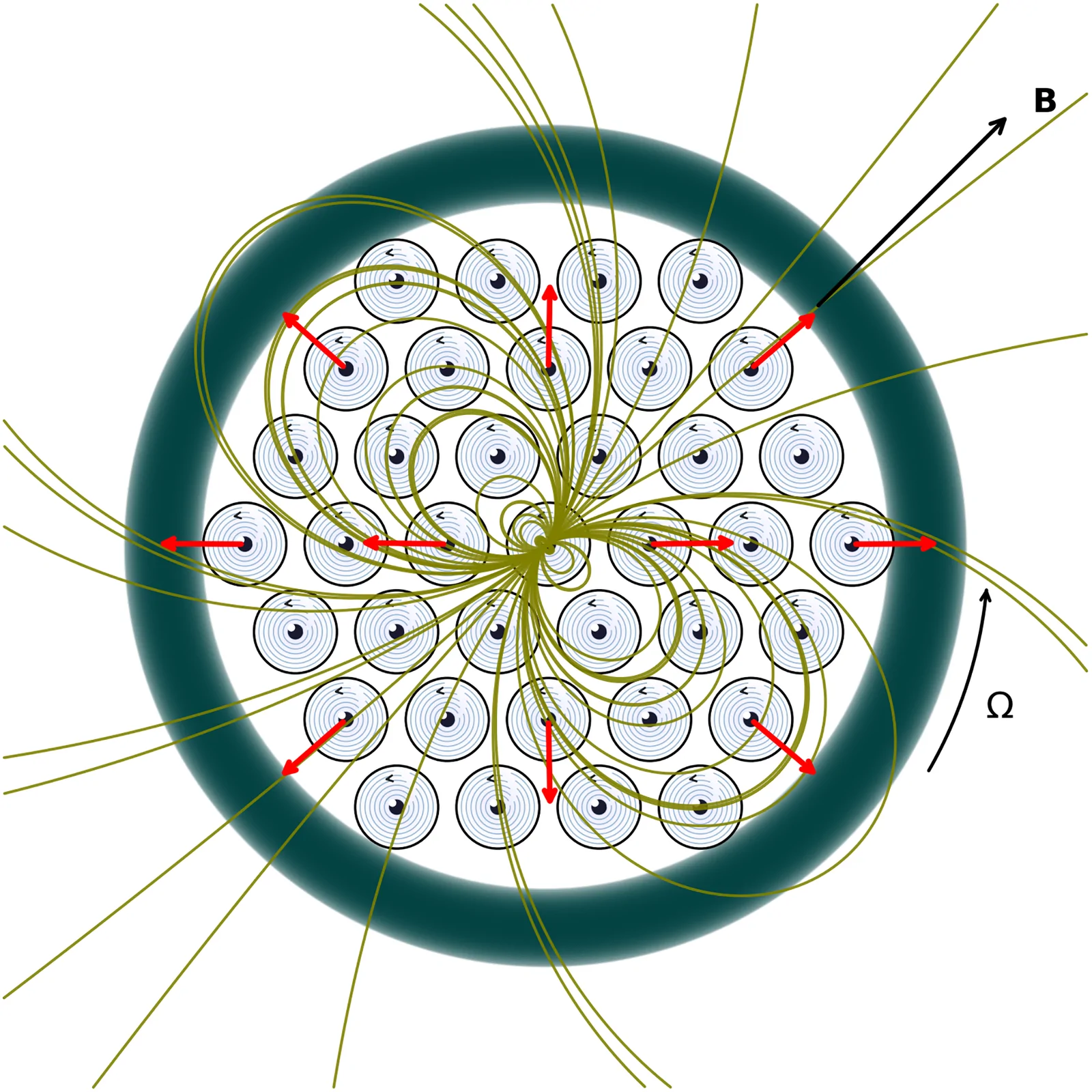
Neutron-star cross section illustrating superfluid vortex lattice, magnetic flux tubes, and spindown-driven vortex motion. A cross section of a neutron star perpendicular to the spin axis, illustrating the triangular lattice of neutron vortices (shown not to scale) that carries the angular momentum of the neutron superfluid. The dipolar magnetic field is also shown, with its axis aligned along the tilted **B** vector, together with the corresponding field lines, which in superconducting proton regions are confined to flux tubes. The red arrows indicate the radial outward motion of neutron vortices in response to the star’s secular spindown.

In general, the rotation and magnetic axes are misaligned, as illustrated in Fig. 5. As the star spins down due to magnetic dipole radiation braking, its rotation frequency decreases. According to Eq. 17, this implies a reduction in the neutron vortex density, which is achieved through an outward radial expansion of the vortex lattice (along the cylindrical radius), as indicated by the arrows in Fig. 5. The corresponding radial velocity of the vortices is

$$v_{r}=-\left(\frac{\dot{\Omega }}{2\Omega }\right)r$$ (19)

which follows from the conservation of the vortex number density $n_{V}$.

Both neutron vortex and proton fluxtube lattices are triangular, with basis lengths

$$d_{n}={\left(\frac{\kappa }{\sqrt{3}\;\Omega }\right)}^{1/2},\;d_{p}={\left(\frac{2\;\phi_{0}}{\sqrt{3}\;B}\right)}^{1/2}$$ (20)

which are of order 10^−4^ and 10^−9^ cm, respectively, for typical neutron-star parameters. These lengths define a mesoscopic scale for neutron-star superfluids, bridging the microscopic scale of the vortex core, set by the coherence length ξ (where the superfluid order parameter vanishes) and the macroscopic scale of the stellar core or crust.

The fermionic vortex core hosts quasiparticle bound states described by Bogolyubov–De Gennes theory (*92*). While in the ^1^*S*_0_ condensate, neutron vortices are well understood, the situation is more complex in the p-wave superfluid, which may exhibit nontrivial topological properties in analogy to the liquid ^3^He in the condensed matter context. For example, Weyl and Majorana fermions can emerge as quasiparticle excitations in neutron ^3^*P*_2_ superfluids (*93*). In addition, there are several branches of bosonic collective modes associated with the spin, in addition to the standard phonon and Higgs modes in s-wave superfluid (*94*–*96*).

It was recognized early on that quantum vortices in neutron ^3^*P*_2_ superfluids can exhibit spontaneous magnetization (*87*–*89*). More recent studies suggest that these vortices may also host Majorana fermions or split into half-quantized non-Abelian vortices (*93*, *97*, *98*). At the interface between the ^3^*P*_2_ and ^1^*S*_0_ superfluid phases, topological surface defects may form (*99*). Furthermore, because the phases of neutron s- and p-wave condensates are different on both sides of their interface, it acts as a Josephson junction, which can excite plasma waves under nonstationary conditions (*100*). The subsequent dissipation of these waves can lead to heating.

Another distinctive feature of the neutron ^3^*P*_2_ phase is its coexistence with the proton ^1^*S*_0_ superconductor. These two condensates are entrained, meaning that the motion of one induces motion of the other. In hydrodynamic terms, their velocities and momenta are linked through a two-by-two entrainment matrix with off-diagonal elements coupling the neutron and proton superflows. A key phenomenological consequence of this entrainment is the appearance of a nonquantized magnetization on neutron vortices (*101*, *102*)—one that exceeds their intrinsic spin-induced magnetization and thereby modifies their coupling to the normal electron and muon fluids (*102*).

Mutual friction arises from the interaction of vortices with the ambient nonsuperfluid components of neutron-star matter and is central to understanding neutron-star rotational dynamics, including glitches and postglitch relaxation (*90*, *103*, *104*). Analogous dissipative mechanisms are well known in other quantum fluids, including both bosonic superfluid ^4^He and fermionic superfluid ^3^He, as well as in ultracold atomic Fermi gases. Neutron stars are nevertheless unique in that they involve the interplay of multiple condensates—most notably a neutron superfluid coexisting with a charged proton superconductor—coupled to a relativistic, highly degenerate electron background under extreme conditions of density and gravity.

For example, electrons scatter off neutron vortex-core quasiparticles via the coupling of their charge –*e* to the neutron magnetic moment. The electron relaxation time in an s-wave neutron superfluid is (*105*)

$$\tau_{\text{eV}}\left[{}^{1}S_{0}\right]=\frac{1.6\times 10^{3}}{\Omega }\frac{\epsilon_{\text{Fe}}^{2}\Delta }{\epsilon_{\text{Fn}}^{2}T}{\left(\frac{\epsilon_{\text{Fn}}}{2m_{n}}\right)}^{1/2}\text{exp}\left(\frac{\epsilon_{1/2}^{0}}{T}\right)$$ (21)

where Δ is the pairing gap, $\epsilon_{Fi}$ are Fermi energies of electrons (e) and (n), $\epsilon_{1/2}^{0}$ is the lowest vortex-core state, and the angular velocity of rotation Ω enters via the vortex density $n_{n}^{\left(V\right)}$. The collision rate $\tau_{\text{eV}}^{-1}$ is seen to be exponentially suppressed at low T.

For a p-wave neutron superfluid, the order parameter is a traceless tensor $A_{\text{μν}}$

$$\begin{array}{c} A_{\text{μν}}=\frac{\Delta }{\sqrt{2}}e^{i\phi }[f_{1}{\hat{r}}_{\mu }{\hat{r}}_{\nu }+f_{2}{\hat{\phi }}_{\mu }{\hat{\phi }}_{\nu }\\ -\left(f_{1}+f_{2}\right){\hat{z}}_{\mu }{\hat{z}}_{\nu }+{\text{if}}_{3}\left(r_{\mu }{\hat{\phi }}_{\nu }+r_{\nu }{\hat{\phi }}_{\mu }\right)] \end{array}$$ (22)

where $f_{1,2,3}\left(r\right)$ describes the radial profile (*89*). p-wave vortices are intrinsically magnetized due to spin-1 Cooper pairs, and electron scattering off them yields a temperature-independent relaxation time (*89*)

$$\tau_{\text{eV}}\left[{}^{3}P_{2}\right]≃\frac{7.91\times 10^{8}}{\Omega }\left(\frac{k_{\text{Fn}}}{{\text{fm}}^{-1}}\right)\left(\frac{\text{MeV}}{\Delta }\right){\left(\frac{n_{e}}{n_{n}}\right)}^{2/3}$$ (23)

where $n_{i}$ are the number densities of species, $k_{\text{Fn}}$ is the neutron Fermi wave vector. Furthermore, the entrainment of proton supercurrent by the neutron vortex circulation induces an effective vortex flux

$$\phi^{*}=k_{\text{ent}}\phi_{0}$$ (24)

where $k_{\text{ent}}$ depends on the proton quasiparticle mass, enhancing the vortex magnetization (*101*, *102*). The associated electron relaxation time is (*102*)

$$\tau_{e\phi }^{-1}=\frac{3\pi }{32}\left(\frac{\epsilon_{\text{Fe}}}{m_{p}c^{2}}\right)\frac{\tau_{0}^{-1}}{k_{\text{eF}}\delta },\;\tau_{0}^{-1}=\frac{2{\mathrm{cn}}_{n}^{\left(V\right)}}{k_{\text{eF}}}\left(\frac{\pi^{2}\phi_{*}^{2}}{4\phi_{0}^{2}}\right)$$ (25)

where $k_{\text{eF}}$ is electron Fermi wave number and $m_{p}$ is the proton mass. This relaxation depends weakly on temperature, reflecting its electromagnetic origin. A full treatment of mutual friction also involves interactions between neutron and proton vortices. If coaxial proton clusters are formed around neutron vortices, the coupling to the electron fluid may increase by orders of magnitude (*106*).

## SUPERFLUIDITY AND ASTROPHYSICS OF COMPACT STARS

### Glitch behavior and recovery

**Fig. 6.**
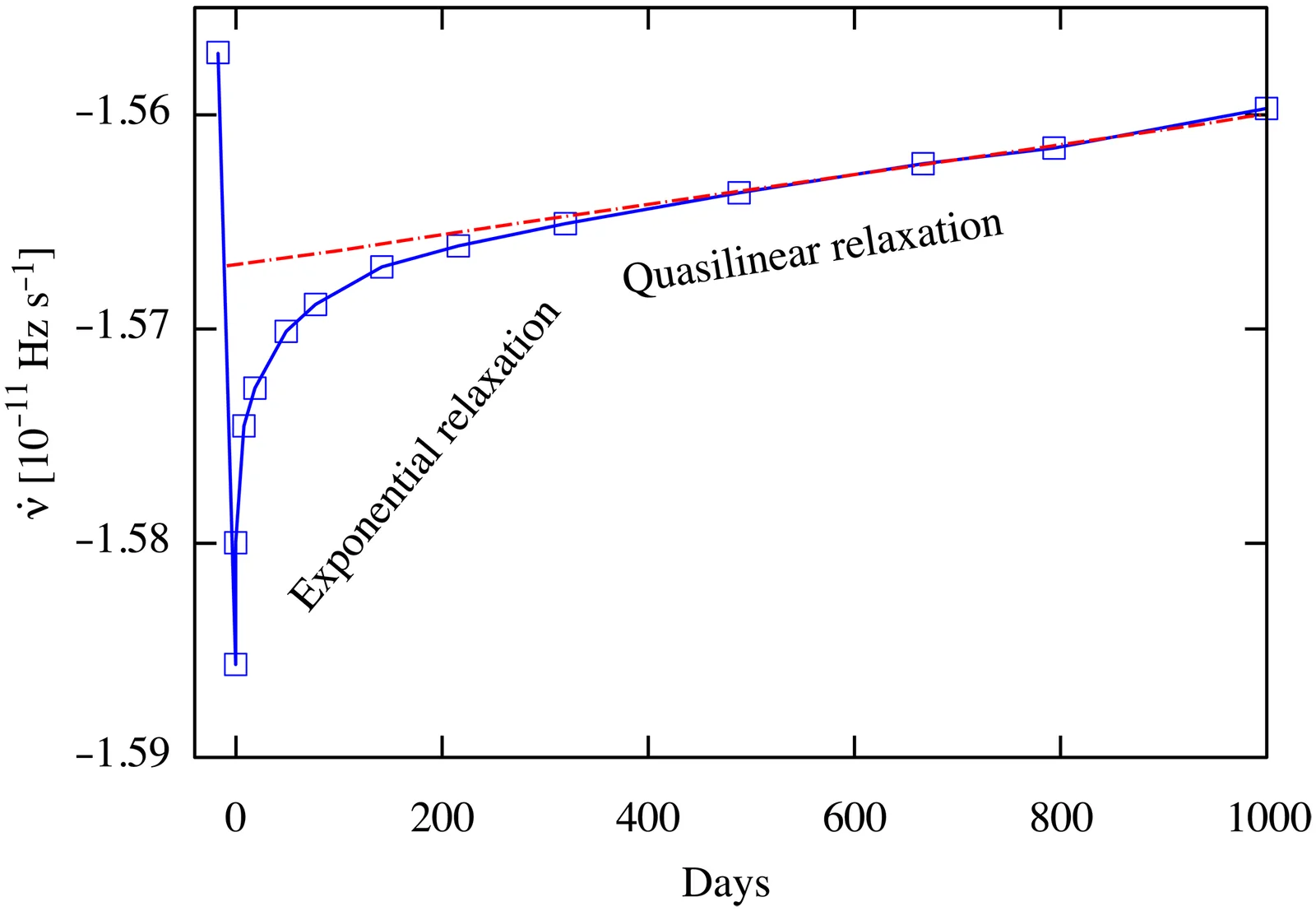
Postglitch evolution of the Vela pulsar’s spindown rate illustrating fast relaxation and extended recovery. Illustration of the glitch and postglitch relaxation in the rotational frequency derivative of the Vela pulsar PSR B0833–45, where the origin of time corresponds to the glitch. The initial, quasi-instantaneous jump in rotation rate is followed by an exponential relaxation over timescales of days to months and subsequently by a quasilinear long-term recovery extending over several years. Data points are adopted from figure 5 in (*109*). The dashed line shows the secular spindown derivative.

Pulsar glitches are sudden jumps in their rotation rate and its derivative that interrupt the otherwise gradual spindown of pulsars, first observed in the Vela pulsar PSR B0833–45. In the 2016 Vela event, the glitch rise was resolved on a pulse-to-pulse basis and constrained to last $\tau_{r}≲16\;s$. The subsequent relaxation of both the rotation frequency and the spindown rate ($\dot{\nu }$), occurring over timescales from minutes to months, points to the presence of a loosely coupled superfluid component within the star (*107*). A representative example of such a glitch and its postglitch evolution in the derivative of the rotation rate of the Vela pulsar is shown in Fig. 6. It is seen that a rapid (exponential) recovery phase is followed by a quasilinear long-term relaxation, which, in general, does not return to the preglitch extrapolated (in the absence of glitch) values, showing permanent frequency offsets.

Glitches are observed to repeat in several young pulsars, most notably in Vela and the Crab, and across the pulsar population, they span a wide range of magnitudes—from small events to so-called giant glitches—highlighting the richness of the underlying physics. From a theoretical perspective, this phenomenology raises several key questions: how and where angular momentum is stored within the star, presumably in a superfluid reservoir; what mechanism enables its rapid release on such short timescales; and what cyclic process can sustain the quasiregular recurrence of glitches.

The dynamics of superfluid interiors provide the most promising framework for explaining glitch behavior. From a theoretical perspective, this involves the motion of quantized vortices in a neutral superfluid—either neutrons in the crust and core or, potentially, superfluid condensates such as the color-flavor–locked (CFL) phase in quark matter. It is now understood that these dynamics operate in two distinct regimes, linear and nonlinear, depending on how the lag between the rotation of the superfluid and that of the normal component evolves under the influence of mutual friction and vortex pinning.

In the linear regime, superfluid hydrodynamics predicts a linear relation between the forces acting on vortices and the relative velocities (lags) between the superfluid, the vortices, and the normal component. In particular, the Magnus force depends on the lag between the superfluid and vortex velocities, while the mutual friction force depends on the lag between the vortex and normal component velocities. Restricting ourselves to the Newtonian case for clarity, the corresponding force balance equation on the vortices can be written as (*108*)

$$\begin{array}{c} \rho_{S}\left[\left(v_{S}+\vec{\nabla }\times \left(\lambda_{V}\varpi \right)-v_{L}\right)\times \kappa \right]\\ -\eta \left(v_{L}-v_{N}\right)+\eta^{\prime }\left[\left(v_{L}-v_{N}\right)\times \varpi \right]=0 \end{array}$$ (26)

where $\rho_{S}$ and $v_{S}$ denote the superfluid density and velocity; $v_{N}$ and $v_{L}$ are the velocities of the normal component and vortices, respectively; κ characterizes the vortex circulation with ϖ=κ/κ; $\lambda_{V}$ is the vortex tension; and $\eta^{\prime }$ term is the transverse friction (Iordanskii) force.

Within the hydrodynamic framework, one can solve for the response of the normal component (the crust together with the charged plasma) to a sudden perturbation. The resulting evolution of the observable rotation frequency and its derivative in a multisuperfluid shell star is given by (*109*, *110*)

$$\begin{array}{c} \nu \left(t\right)=\nu_{0}-\frac{\nu_{0}}{\tau_{0}}t\\ -\sum_{i}\frac{I_{Si}}{I_{c}}\left(\frac{\nu_{0}}{\tau_{0}}\tau_{i}-\Delta \nu_{\text{Si}}\right)\left(1-e^{-t/\tau_{i}}\right) \end{array}$$ (27)

$$\dot{\nu }\left(t\right)=-\frac{\nu_{0}}{\tau_{0}}-\sum_{i}\frac{I_{Si}}{I_{c}}\left(\frac{\nu_{0}}{\tau_{0}}\tau_{i}-\Delta \nu_{Si}\right)\frac{e^{-t/\tau_{i}}}{\tau_{i}}$$ (28)

where $\nu_{0}$ is the pulsar’s rotation frequency at the reference time (typically just before the glitch) and $\tau_{0}$ is the characteristic spindown timescale, defined through the external torque acting on the star. Fitting these expressions to observational data allows one to extract the characteristic coupling timescales $\tau_{i}$ and the associated superfluid moments of inertia $I_{Si}$, given the moment of inertia of the normal component $I_{c}$, which to a good approximation, is equal to the total moment of inertia of the star.

The relaxation timescale appearing in Eqs. 27 and 28, which characterizes the exponential decay of the postglitch response, can be related to the parameters entering the force balance Eq. 26 as (*106*, *111*)

$$\tau =\frac{1}{2\Omega_{s}\left(0\right)}\left(\frac{\eta }{\rho_{S}\kappa }+\frac{\rho_{S}\kappa }{\eta }\right)$$ (29)

where, for simplicity, we have suppressed the index i labeling different superfluid regions. Here, $\Omega_{s}\left(0\right)$ is the initial angular velocity of the superfluid, and η denotes the mutual friction coefficient describing the interaction between vortices and the normal component (predominantly ultrarelativistic electrons). In writing this expression, we neglected the transverse friction term by setting $\eta^{\prime }=0$. Notably, the dynamical relaxation time has a nontrivial dependence on the viscous friction coefficient η. It takes on large values in two limiting cases $\eta \ll \rho_{S}\kappa$ and $\eta \gg \rho_{S}\kappa$, corresponding to the weak- and strong-coupling limits between the vortices and the normal component. The minimum of $\tau_{d}$ is achieved, when $\eta =\rho_{S}\kappa$ with $\tau_{d}\left(\text{min}\right)=\Omega_{s}^{-1}\left(0\right)$, which sets the absolute minimum on the relaxation time.

Although the weak-coupling regime was initially invoked to explain glitches—attributing the long relaxation times to weak coupling between vortices and the electron fluid (*107*)—an alternative picture emerged in the 1970s, inspired by the resistive state of type-II superconductors. In that context, for small driving currents, a vortex lattice responds via thermally activated motion, leading to an exponential creep of vortices through pinning centers.

**Fig. 7.**
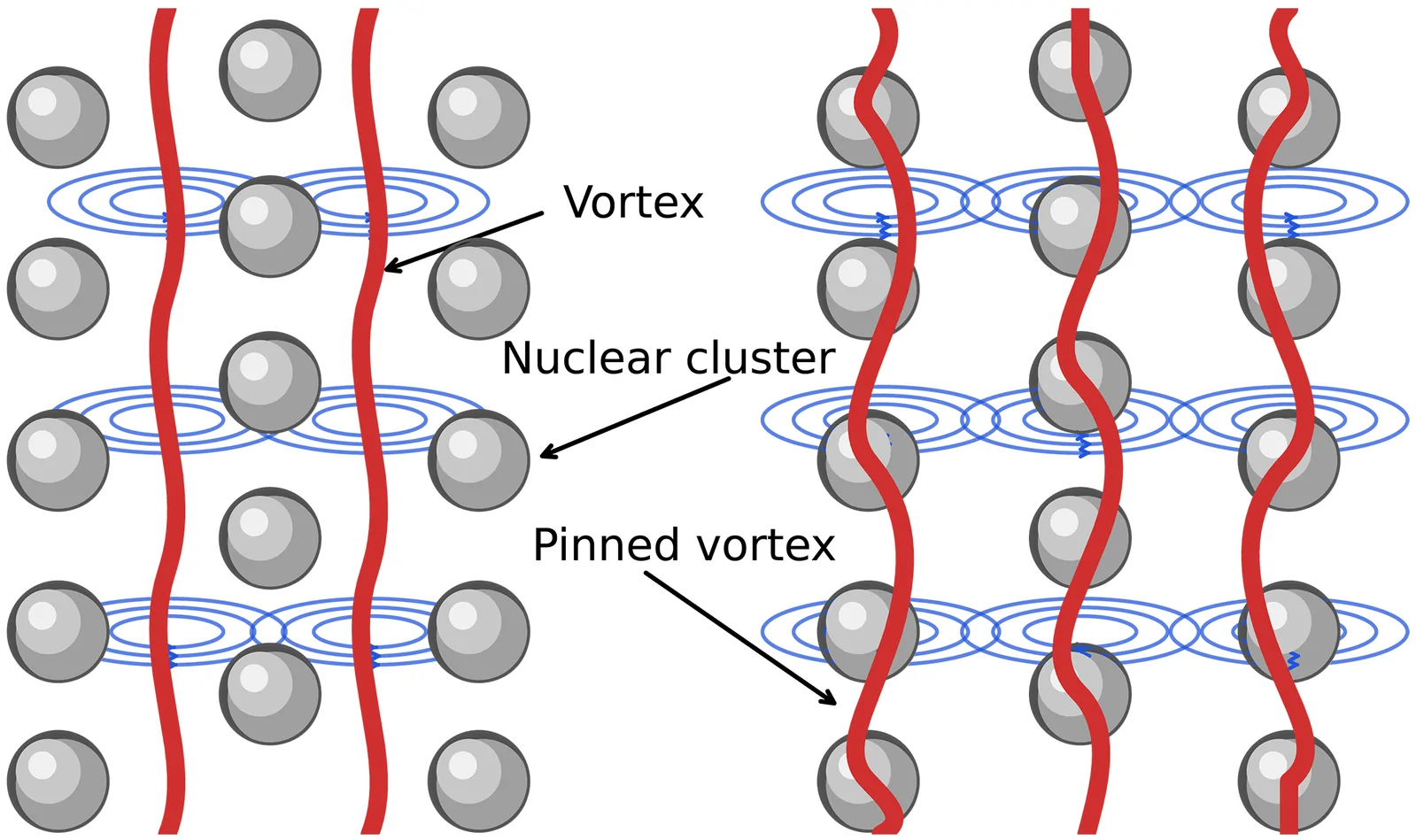
Two regimes of vortex dynamics in the neutron-star crust: free motion and thermally activated creep of pinned vortices. Illustration of two distinct regimes of vortex dynamics in the neutron-star crust: On the left, vortices move freely through the nuclear lattice (the unpinned, linear regime), while on the right, vortices are pinned to lattice nuclei. In the latter case, vortex creep proceeds via thermally activated, jump-like motion of vortices from one pinning center to another.

Neutron-star crusts host both a lattice of quantized vortices and a lattice of nuclear clusters, which coexist and interact with each other. The nuclear clusters can act as pinning centers because certain configurations of a vortex relative to a cluster minimize the total energy of the system. This leads to the possibility of vortex pinning, where vortices become temporarily anchored to the crustal lattice. Figure 7 illustrates two limiting regimes: On the left, vortices move freely under the action of forces in the linear (unpinned) regime, while on the right, vortices are pinned to the nuclear lattice.

Anderson and Itoh (*112*) proposed that glitches arise from the sudden unpinning of a superfluid component that is otherwise decoupled from the observable crust, resulting in a rapid transfer of angular momentum. In more detail, before a glitch, the crust spins down under the action of an external torque, while the superfluid component remains partially decoupled, leading to a gradual buildup of a rotational lag ΔΩ between the two components. When this lag exceeds a critical threshold, $\Delta \Omega ≳{\Delta \Omega }_{c}$, vortices that were previously pinned to the crust suddenly unpin. This triggers the glitch itself, which occurs on very short timescales (seconds or less) and involves a rapid transfer of angular momentum from the superfluid to the crust, resulting in $\Omega_{c}$ increasing and $\Omega_{s}$ decreasing. Following the glitch, the system undergoes a relaxation phase over timescales ranging from minutes to months, during which the superfluid and normal components gradually recouple through mutual friction or vortex creep. This recovery is typically well described by exponential relaxation with characteristic timescales $\tau_{i}$.

In crustal vortex creep models, the postglitch relaxation is governed by thermally activated vortex motion [see (*113*) and references therein], with a radial velocity

$$v_{r}\approx v_{0}\text{exp}\left(-E_{a}/k_{B}T\right)$$ (30)

where $v_{0}∼10^{7}$ cm s${}^{-1}$ and the activation energy is given by $E_{a}\approx E_{p}\left(1-\Delta \Omega /{\Delta \Omega }_{c}\right)$. Here, ΔΩ denotes the lag between the superfluid and the crust, ${\Delta \Omega }_{c}$ is the critical lag for unpinning, and $E_{p}$ is the pinning energy. The evolution of the observable crustal angular velocity $\Omega_{c}$ then follows

$$I_{c}{\dot{\Omega }}_{c}=N_{\text{ext}}+\sum_{i}I_{s}^{i}\frac{2\Omega_{s}^{i}}{r}v_{r}^{i}$$ (31)

where $I_{c}$ and $I_{s}^{i}$ are the moments of inertia of the normal component and the ith superfluid component, respectively; $\Omega_{s}^{i}$ is the angular velocity of that component; r is the cylindrical radius; and $N_{\text{ext}}$ is the external torque.

A generic consequence of angular momentum conservation during a glitch

$$I_{c}\Omega_{c}+\sum_{i}I_{s}^{i}\Omega_{s}^{i}=\text{const}$$ (32)

is that a sudden exchange of angular momentum between the superfluid interior and the solid crust satisfies $I_{c}{\Delta \Omega }_{c}+\sum_{i}\left(I_{s}^{i}{\Delta \Omega }_{s}^{i}\right)=0$. The observed amplitudes of large glitches—such as those in the Vela pulsar—require that a superfluid component with a fractional moment of inertia of order $\sum_{i}I_{s}^{i}/I∼10^{-2}$ participates in the dynamics, where I is the total stellar moment of inertia. Furthermore, analyses of Vela’s glitches over several decades indicate that these large events require at least ≳1.4% of the star’s moment of inertia to be involved, thereby setting a lower bound on the superfluid reservoir (*114*).

Although the crystalline lattice of the inner crust provides a natural site for vortex pinning, it also introduces band-structure effects: Neutrons occupy Bloch states rather than moving freely, which can substantially reduce the effective superfluid fraction (*115*). This finding implies that crust-only models are insufficient because the moment of inertia associated with the crustal superfluid is significantly smaller. More recent work indicates that allowing interband transitions can restore the superfluid density to values approaching those of quasifree neutrons (*116*). For complementary low-dimensional analyses, see (*117*).

Together, these results suggest that the superfluid component responsible for large glitches may extend beyond the crust, including part of the outer core, where neutron pairing in the ^3^*P*_2_ channel provides an additional reservoir of angular momentum required to reproduce the observed glitch magnitudes. If the core were devoid of proton flux tubes, then the magnetization of p-wave neutron vortices, induced by entrainment, would couple the core superfluid to the normal component on timescales much shorter than those inferred from postglitch relaxation (*102*).

However, the presence of proton flux tubes in the superconducting core can significantly modify this picture, as they may act as pinning centers for neutron vortices, thereby adding to the mutual coupling between the superfluid and the normal component (*90*). As illustrated in Fig. 5, the motion of neutron vortices can be hindered through their interaction with the flux-tube array. To some extent, this situation is analogous to the pinning of neutron vortices to the crustal lattice, although it occurs in the core and involves magnetic structures rather than nuclear clusters.

Core-based glitch models have been developed within the vortex-cluster framework, in which each neutron vortex is surrounded by a coaxial, dense mesh of proton flux tubes whenever the entrainment-induced magnetic field of the vortex exceeds the lower critical field for flux-tube formation (*106*). In this regime, the electron scattering off vortex clusters, dressing each neutron vortex, leads to strong coupling limit ($\eta \gg \rho_{S}\kappa$), consequently, to long dynamical relaxation timescales consistent with observations (*106*, *118*). In the presence of toroidal fields, neutron vortex creep against the equatorially distributed flux-tube arrays in the core can provide an alternative mechanism for glitch recovery (*119*).

The glitch mechanism itself may involve a combination of vortex dynamics and crust quakes, the latter relieving stresses that accumulate as the star decelerates. In self-organized criticality models, avalanches of vortex unpinning can trigger glitches, producing scale-invariant, power-law distributions of glitch sizes and exponential waiting-time distributions—features consistent with most pulsars except for Vela and PSR J0537–6910, which exhibit quasiperiodic behavior (*104*). Small glitches may be suppressed when the number of freely moving vortices is limited or when mutual friction is reduced.

Alternative or complementary triggers include crustal fractures and hydrodynamical instabilities within the superfluid interior and phenomena at the crust-core interface [for a review, see (*103*)]. In addition, both classical and quantum (superfluid) turbulence may play an important role in the nonlinear dynamics of the superfluid and in the coupling between its components (*120*).

Poli *et al*. (*121*) leveraged the analogy between neutron stars and dipolar supersolids to gain full experimental access to the system. In particular, changes in the moment of inertia arising from internal dynamics can now be accurately measured in the laboratory, providing a clear pathway to modeling neutron-star glitches in controlled experimental settings. This approach opens opportunities to investigate the microscopic mechanisms underlying glitches and to directly connect observed dynamical signatures to internal vortex and lattice dynamics.

Marmorini *et al*.(*122*) attribute neutron-star glitches to quantum vortex networks formed at the interface between two superfluid phases in the core: a p-wave neutron superfluid in the inner core and an s-wave neutron superfluid in the outer core. In this configuration, each integer vortex in the s-wave superfluid connects to two half-quantized vortices in the p-wave superfluid via topological structures known as “boojums” (*122*). The crust-core interface may act as a trigger for glitches because it can serve as a barrier for proton flux tubes dressing neutron vortices, preventing their continuous annihilation. Furthermore, on secular timescales, the interface between the s-wave and p-wave superfluids can radiate energy due to the time-dependent Josephson current induced by vortex plus flux-tube motion. This Josephson current arises because the phases of s-wave and p-wave superfluids differ on both side of their interface (*100*).

The microphysics of pairing plays a central role in determining which regions of a neutron star participate in glitch dynamics. Because superfluidity (and superconductivity) is a prerequisite for vortex-mediated angular momentum transfer, any region where pairing is suppressed is effectively excluded from contributing to glitches. For example, strong magnetic fields may quench proton superconductivity in parts of the core, thereby modifying or eliminating flux-tube–vortex interactions and altering the coupling between components. At the same time, vortex creep models are directly sensitive to the underlying pairing properties, as the pinning energies and mobility of vortices depend on the magnitude of the pairing gap.

If the neutron pairing gap is reduced or suppressed in the crust, the available superfluid reservoir there may be insufficient to account for observed glitch amplitudes, implying that the core superfluid must play a partial or even dominant role in glitch activity and postglitch relaxation. Pairing in neutron stars can, to a good approximation, be described within the BCS (weak-coupling) regime. A strong-coupling regime—associated with very low densities and large pairing gaps—is only marginally realized near the low-density boundary between the inner and outer crust and does not play a significant role in the bulk of the star.

### Long-term variabilities

Apart from glitches, neutron stars may exhibit complex long-term rotational variability arising from the interplay between their solid crust and superfluid interior. The classical-like free precession, familiar from rigid-body dynamics, involves the slow rotation of the symmetry axis about the total angular momentum vector. In stars with weakly coupled superfluids, this mode can persist for months or years with the frequency essentially that of classical precession

$$\omega_{\text{slow}}≃\epsilon \Omega$$ (33)

ϵ is the eccentricity, and Ω is the stellar rotation rate. Superimposed on this is a fast precession mode associated with the independent motion of the neutron superfluid. Its frequency scales as

$$\omega_{\text{fast}}\propto \frac{I_{s}}{I_{n}}\Omega$$ (34)

This mode arises because the superfluid vortices introduce additional degrees of freedom relative to a classical rigid body (*123*–*125*). Tkachenko waves provide another source of long-term variability in neutron stars (*126*, *127*). The Tkachenko mode frequency in the nondissipative limit is given in units of 2Ω by

$$p_{T}=\pm \sqrt{\text{cos}\theta +g}$$ (35)

where θ is the angle between the rotation (spin) axis—i.e., the vortex-line direction—and the wave vector k and $g\equiv \left(k^{2}/4\Omega^{2}\rho_{n}\right)$$\left[\mu_{n}-\text{cos}\theta \left(\mu_{n}-2\Omega \lambda_{V}\right)\right]$, where $\rho_{n}$ is the superfluid density, $\lambda_{V}$ is the vortex tension, and $\mu_{n}=\rho_{n}\hbar \Omega /8m_{n}$ is the shear modulus of the triangular vortex lattice. It is useful to consider the limiting cases of the angular dependence of the Tkachenko modes, specifically in the case of parallel propagation (θ=0)

$$p_{T}=\pm {\left(1+\frac{k^{2}}{2\Omega \rho_{n}}\lambda_{V}\right)}^{1/2}$$ (36)

showing that the mode is dominated by vortex-line tension. In the case of perpendicular propagation (θ=π/2)

$$p_{T}=\pm \frac{k}{2\Omega }\sqrt{\frac{\mu_{n}}{\rho_{n}}}$$ (37)

which gives the standard Tkachenko elastic scaling.

For a general θ, the cosθ term acts as a geometric “gap,” while the $k^{2}$ term encodes lattice elasticity and vortex tension. Physically, this reflects the crossover from transverse shear oscillations of the vortex lattice (for k⊥Ω) to longitudinal distortions along vortex lines (for k∥Ω), which is exactly what one expects in a rotating superfluid or supersolid.

We note that in a uniformly rotating system, the full low-frequency spectrum contains in addition to Tkachenko modes (originating from elastic shear deformations of the vortex lattice, with restoring forces provided by the lattice shear modulus and vortex line tension), the inertial modes, which exist without a vortex lattice. They are restored purely by the Coriolis force and have frequencies $p_{I}=\pm \text{cos}\theta$ in units of 2Ω. These modes are nondispersive, i.e., independent of k at leading order, and vanish for k⊥Ω.

The influence of mutual friction on Tkachenko modes in liquid ^4^He was first studied in (*128*). This framework was later generalized to include the effects of shear viscosity in the normal component and applied to neutron stars’ interiors in (*127*) using Newtonian dissipative superfluid hydrodynamics. In that context, Tkachenko modes were found to have characteristic periods ranging from months to years, depending on the rotation rate Ω, naturally matching the long-term periodicities observed in pulsars. The observable variability is further shaped by mutual friction between vortices and the normal fluid, which damps both precessional and Tkachenko motions.

In the weak-coupling regime, one expects combined signatures of long postglitch relaxation times together with the possibility of quasifree precession. In contrast, in the strong-coupling limit, precession is strongly suppressed due to efficient coupling between the superfluid and the normal component, although long-term glitch relaxation can still persist. Tkachenko modes—low-frequency oscillations of the vortex lattice—are likewise sensitive to the dissipative properties of the medium: They are significantly modified by mutual friction and shear viscosity (*127*), while bulk viscosity does not enter the characteristic equations governing these modes and therefore has no direct impact on their dynamics.

In our Review, we adopted a Newtonian multifluid hydrodynamic framework, which captures the essential features of superfluid dynamics, vortex motion, and the coupling between superfluid and normal components relevant for glitch phenomena. This approach provides a transparent and tractable description of the underlying physics. However, fully relativistic formulations of superfluid hydrodynamics do exist (*129*, *130*). More recently, frameworks (*131*–*133*) have been developed to account for finite temperature and/or multiple components, incorporating vortices, flux tubes, and mutual friction; these formulations link microscopic physics to macroscopic dynamics, keeping the general-relativistic framework intact on all scales.

## SPIN, SUPERFLUIDITY, AND VORTICITY IN QUARK MATTER

### Color superconductivity in quark matter

Building on the discussion of pairing in nucleonic matter, similar pairing phenomena are expected to occur in deconfined quark matter at sufficiently high densities, where quarks form Cooper pairs due to attractive channels in the underlying QCD interaction; for a broad overview, see (*134*). This leads to the emergence of color-superconducting phases, whose structure depends on the number of quark flavors participating, their masses, and the mismatch between their Fermi surfaces imposed by beta equilibrium. The simplest and most robust phase at intermediate densities is the two-flavor color-superconducting (2SC) phase (*135*), in which up and down quarks pair in a color-antitriplet channel, leaving one color unpaired.

At asymptotically high densities, where the strange quark becomes effectively light and Fermi momenta are nearly equal, the CFL phase is expected to be the ground state (*136*). In this phase, all three flavors participate symmetrically in pairing, leading to a fully gapped spectrum and peculiar properties such as the absence of electrons (in the simplest approximation). Kaon condensation can reintroduce electrons once stress due to finite strange quark mass or charge neutrality drives the system away from exact symmetry, leading to the breaking of the corresponding flavor symmetries and the onset of meson-condensed phases (*137*, *138*).

At densities relevant for neutron stars, however, these idealized pairing patterns are further modified by the stress induced by finite strange quark mass and chemical potential differences between flavors. This can give rise to phases with broken spatial symmetries, such as Fulde-Ferrell–type phases where Cooper pairs carry nonzero momentum (*139*), or phases with deformed Fermi surfaces (*140*).

These possibilities highlight that, much similar to nucleonic matter, the pairing structure in quark matter is rich and sensitive to microscopic conditions. The resulting phases can significantly affect transport and neutrino emission and therefore play an important role in determining the observable properties of hybrid stars (*134*, *141*).

Somewhat similarly to nucleonic matter, in color-superconducting phases of dense quark matter, magnetic fields couple to quark spins and electric charges, leading to Fermi surface mismatches between pairing species and thereby suppressing Cooper pairing via a Pauli paramagnetic-type mechanism; in this context, the magnetic field and the finite strange quark mass play competing and qualitatively similar disruptive roles. An additional parallel with nucleonic matter arises from the coupling of charged quarks to the magnetic field, which induces Landau quantization and modifies the pairing structure. These effects are particularly relevant for phases such as 2SC and CFL, where it has been shown that magnetic fields can significantly alter the gap structure, induce anisotropies, and even lead to the emergence of novel pairing patterns. In particular, studies of the CFL phase in a magnetic field demonstrate the splitting of pairing gaps and the appearance of magnetically modified CFL phase, while analyses of more general quark matter phases indicate that magnetic fields can favor pseudoscalar condensates and modify the phase diagram in a nontrivial way (*142*, *143*).

In color-superconducting quark matter, the response to magnetic fields is more subtle than in conventional superconductors due to the mixing between the electromagnetic gauge field and a component of the gluon field. In particular, the diquark condensate breaks the original $U{\left(1\right)}_{\text{em}}$ symmetry, but leaves unbroken a rotated $U{\left(1\right)}_{\tilde{Q}}$ symmetry corresponding to a linear combination of the photon $A_{\mu }$ and the eighth gluon $G_{\mu }^{8}$. The resulting massless gauge field

$$A_{\mu }^{\tilde{Q}}=A_{\mu }\text{cos}\theta -G_{\mu }^{8}\text{sin}\theta$$ (38)

defines the so-called rotated photon, while the orthogonal combination

$$A_{\mu }^{X}=A_{\mu }\text{sin}\theta +G_{\mu }^{8}\text{cos}\theta$$ (39)

acquires a Meissner mass. The mixing angle θ is determined by the electromagnetic and strong coupling constants, typically satisfying tanθ∼e/g≪1, so that the rotated photon is predominantly electromagnetic (*144*).

As a consequence, magnetic fields associated with the unbroken $U{\left(1\right)}_{\tilde{Q}}$ can penetrate the medium, while those coupled to the massive X field are screened. In the 2SC phase, this leads to partial penetration of magnetic flux and a modified Meissner effect rather than complete expulsion as in ordinary superconductors. In the CFL phase, the response to magnetic fields is governed by a similar mixing between the electromagnetic gauge field and a gluon but with an important difference arising from the fully symmetric participation of all three flavors in pairing. A key distinction of the CFL phase is that all quark quasiparticles are neutral under the rotated charge $\tilde{Q}$. As a result, the medium is an insulator with respect to $U{\left(1\right)}_{\tilde{Q}}$, and no electrons are required to ensure charge neutrality in the idealized limit. Magnetic fields associated with the massless rotated photon can therefore penetrate the CFL phase essentially unimpeded, while the orthogonal X component is screened over a short length scale. Consequently, the CFL phase does not exhibit a conventional Meissner effect for ordinary magnetic fields but instead allows partial penetration determined by the projection onto the unbroken $U{\left(1\right)}_{\tilde{Q}}$ sector.

### Quantum vorticity in quark matter

If the color-superconducting phase is of type II with respect to the X field, the corresponding lower critical field is very large, $H_{c1}^{\left(X\right)}∼10^{17}G$ (*144*). Nevertheless, this does not preclude the formation of color-magnetic flux tubes. During the transition into the superconducting phase, X flux can become trapped between growing superconducting domains and subsequently compressed. As these domains merge, the local magnetic field in the remaining normal regions can exceed $H_{c1}^{\left(X\right)}$, leading to their fragmentation into Abrikosov-like flux tubes that carry quantized X flux.

In contrast, in the CFL phase, all quark quasiparticles are neutral under the rotated charge $\tilde{Q}$, so that the medium behaves as an insulator with respect to the unbroken $U{\left(1\right)}_{\tilde{Q}}$, and magnetic fields associated with the rotated photon can penetrate essentially unimpeded, while the orthogonal X component remains screened. Futhremore, in the CFL phase, breaking of baryon number implies superfluidity, so vortices must appear under rotation; however, the energetically favored defects are not simple $U{\left(1\right)}_{B}$ vortices, as discussed in the nucleonic case, but non-Abelian semisuperfluid vortices that carry both fractional circulation and color-magnetic flux (*145*). The CFL matter supports semisuperfluid strings and flux tubes, and later work clarified that the minimal CFL vortices are (1/3)-quantized objects with long-range repulsion, suggesting the formation of a vortex lattice. These vortices also support nontrivial low-energy degrees of freedom in their cores, including orientational modes and, in some treatments, gapless fermionic modes.

Vorticity has been experimentally observed in the quark-gluon plasma created in relativistic heavy-ion collisions (*146*); however, unlike the quantized vortices in superfluids, vorticity in the quark-gluon plasma is a property of a relativistic fluid without long-range phase coherence and therefore differs fundamentally in its microscopic origin despite some formal analogies.

An important question for neutron stars is how such quark vortices connect to hadronic vortices across a hadron-quark interface. Earlier work on colorful boojums showed that junction structures can arise when neutron and proton vortices from hadronic matter penetrate a CFL core, with the boojum acting as the interface where hadronic circulation is redistributed into color-carrying CFL vortices (*147*). Related studies (*148*) discuss vortex continuity and argue that, in crossover scenarios, hadronic vortices may connect to non-Abelian CFL vortices. Alford *et al*. (*149*) showed that in three-flavor symmetric matter, a singly quantized hadronic vortex can connect smoothly to a singly quantized non-Abelian CFL vortex, so a boojum is not always required. Because CFL vortices carry color-magnetic flux, they are often described as simultaneously superfluid and magnetic defects (*150*). Together, the vortex sector of quark matter is likely to play a central role in determining the rotational and magnetic properties of hybrid stars. Together, the vortex sector of quark matter is likely to be central for the rotational, magnetic, and transport properties of hybrid stars. In particular, in the 2SC phase, color-magnetic flux tubes interact with gapless fermionic excitations via the Aharonov-Bohm scattering (*144*), leading to a significant drag force that can strongly influence their dynamics and mobility and thereby affect magnetic field evolution and transport properties in the stellar core.

In quark matter phases, there is a close analogy to vortex pinning in neutron-star hadronic phases, where vortices interact with the nuclear lattice in the crust or with proton flux tubes in the core. It turns out that in phases with broken spatial symmetries, such as Fulde-Ferrell or more general crystalline phases (*151*), the structure of vortices becomes even more intricate. In these phases, the pairing gap varies periodically in space, and the order parameter carries finite momentum. Consequently, vortices are embedded in an inhomogeneous background, leading to anisotropic cores and modified tension and mobility. The periodic modulation of the condensate can act as an intrinsic pinning lattice for vortices, while the presence of rotated electromagnetism implies that magnetic flux and superfluid circulation may be intertwined in a nontrivial way. These features suggest that quark matter phases with broken spatial symmetries can exhibit qualitatively new vortex and flux-tube dynamics, with potential implications for angular momentum transport and glitch phenomena in hybrid stars.

## CONCLUSIONS

We reviewed selected aspects of the interior physics of compact stars, with particular emphasis on the microscopic and macroscopic manifestations of spin, magnetic fields, and nucleonic superfluidity and superconductivity. Spin plays a fundamental role in the stability of neutron stars, which are supported by the degeneracy pressure of fermionic matter. In describing the EoS of dense matter, we adopted a phenomenological (metamodeling) approach based on an expansion of the nuclear-matter energy density around the isospin-symmetric limit and near saturation density. This framework enables the generation of families of EoSs that can be used in the general-relativistic stellar-structure equations to determine global stellar observables such as mass, radius, and moment of inertia.

We reviewed the current state of observational constraints and discussed prospects for improving them through forthcoming multimessenger observations. We also examined the influence of magnetic fields on the EoS of dense matter, noting that significant modifications require extremely strong fields and may involve the emergence of spin-polarized or ferromagnetic phases—possibilities that remain highly uncertain at the densities relevant to neutron-star interiors.

At the mesoscopic scale, the interplay between superfluid and superconducting components gives rise to a rich variety of vortex and flux-tube phenomena. Neutron superfluid rotation proceeds through the formation of a quantized vortex lattice, while protons form a type-II superconductor characterized by critical fields consistent with those expected in neutron-star cores. Pinning interactions—between vortices and nuclei in the crust or between vortices and magnetic flux tubes in the core—provide a natural mechanism for understanding the nonstationary rotational dynamics of neutron stars.

Outstanding issues include the internal structure of ^3^*P*_2_-^3^*F*_2_ vortices, the possible emergence of type-I superconductivity in the deep core, the configuration and dynamics of proton flux tubes, the magnitude of mutual friction coefficients in different pairing regimes, and the extent of crustal entrainment. The dynamics at the mesoscopic scale connects directly to macroscopic models of pulsar glitches and postglitch relaxation, which remain unresolved regarding the precise location and extent of the superfluid regions responsible for short- and long-term relaxation processes. While several generic glitch triggers are qualitatively understood, a quantitative description remains incomplete. Major open problems include identifying the location and nature of glitch activity (crustal, core, or mixed), determining the components responsible for postglitch recovery, and explaining the observed diversity of glitch behavior.

We also reviewed the possible role of deconfined quark matter and color superconductivity in the inner cores of hybrid stars. The rich pairing structure of dense QCD gives rise to a variety of superconducting phases, each with distinct transport and magnetic properties. In particular, color-superconducting phases support novel vortex configurations, including color-magnetic flux tubes in the 2SC phase and non-Abelian semisuperfluid vortices in the CFL phase, which intertwine superfluid circulation with rotated electromagnetic flux. In the CFL-like phases, these structures may play an important role in the rotational dynamics of hybrid stars, providing additional channels for angular momentum transfer and mutual friction, through such mechanisms as scattering of electrons and quarks off color-magnetic defects via Aharonov-Bohm interactions. At the same time, the modified electromagnetic response of color-superconducting matter, governed by rotated gauge fields, may affect the evolution and topology of magnetic fields in the stellar core. While these possibilities remain model dependent and subject to significant uncertainties in the QCD phase diagram at neutron-star densities, they underscore the potential of quark matter to leave observable imprints on both the rotational and magnetic behavior of compact stars.

In conclusion, ongoing and forthcoming multimessenger observations—across the electromagnetic spectrum and through gravitational-wave detections—are expected to provide crucial insights into the structure and dynamics of neutron stars. These observations will offer essential feedback for refining theoretical models, linking microscopic nuclear interactions to macroscopic astrophysical observables and thereby deepening our understanding of strongly interacting matter under extreme conditions.
